# Tailoring Antioxidant Activities: Metal-Type Dependent, Highly Active SOD or Catalase Mimetics

**DOI:** 10.1021/acs.inorgchem.5c02973

**Published:** 2025-09-09

**Authors:** Álvaro Martínez-Camarena, Pablo Navarro-Madramany, Carmen E. Castillo, Antonio Doménech-Carbó, Manuel G. Basallote, Peter Faller, Enrique García-España

**Affiliations:** † ICMol, Departament de Química Inorgànica, Universitat de València, C/Catedrático José Beltrán 2, 46980 Paterna, Spain; ‡ Institut de Chimie, UMR 7177, Université de Strasbourg, CNRS, 4 Rue Blaise Pascal, 67000 Strasbourg, France; § MatMoPol Research Group, Departamento de Química Inorgánica, Facultad de Ciencias Químicas, Universidad Complutense de Madrid, Avda. Complutense s/n, 28040 Madrid, Spain; ∥ Departamento de Ciencia de los Materiales e Ingeniería Metalúrgica y Química Inorgánica, Facultad de Ciencias, Instituto de Biomoléculas (INBIO), 16727 Universidad de Cádiz, Puerto Real 11510, Cádiz, Spain; ⊥ Departament de Química Analítica, Universitat de València, C/Dr Moliner s/n, 46100 Burjassot, Spain; # Institut Universitaire de France (IUF), 1 rue Descartes, 75231 Paris, France

## Abstract

The failure of the therapeutic administration of superoxide dismutase (SOD) and catalase (CAT) enzymes to prevent oxidative stress has fostered the development of metal complexes that are capable of mimicking their activity. In the present work, two new pyridine azacyclophane ligands capable of coordinating Cu^2+^ and Fe^2+^ to give rise to mimetics with high activities toward disproportionation of the superoxide anion or hydrogen peroxide, depending on the metal ion, have been prepared. Although the Cu^2+^ complexes have some of the highest SOD activities reported to date, they are completely inactive toward H_2_O_2_ disproportionation. In contrast, the Fe^2+^ complexes catalyze the disproportionation of H_2_O_2_ without showing any catalytic SOD activity. Therefore, the type of antioxidant activity of these macrocycles is dictated by the nature of the metal ion, which represents a new approach to the development of potentially useful mimetics.

## Graphical abstract

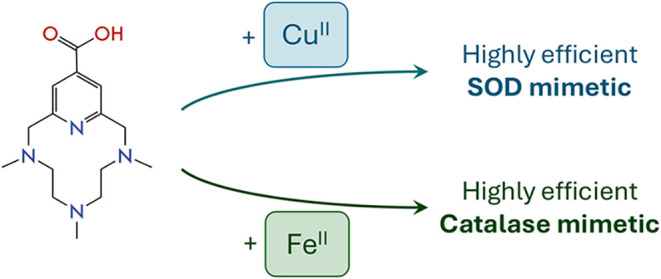

## Introduction

Although dioxygen is essential for aerobic organisms, under certain conditions, it can be transformed into potentially harmful reactive oxygen species (ROS), such as superoxide anion and hydrogen peroxide. On their own, ROS are not inherently problematic, partly because aerobic organisms have extremely efficient defense systems for their removal, such as the enzymes superoxide dismutases (SODs) and catalases (CATs),^1,2^ and partly because these same organisms can use ROS to their own benefit, as they are involved in the cellular signaling pathways^3^ as well as in the elimination of pathogens in macrophages.^4^ The risk arises when there is an unregulated excess of these species, i.e., when their generation exceeds the capacity of the metabolism to remove or handle them. This imbalance results in oxidative stress.

Oxidative stress is a pathogenic condition associated with a range of detrimental health issues, including cardiovascular diseases,^5,6^ chronic inflammation,^7^ type 2 diabetes,^8^ cancer,^9^ aging,^10^ and neurodegenerative disorders,^11^ such as Huntington’s,^12,13^ Parkinson’s,^14,15^ and Alzheimer’s diseases.^16,17^ Among the different pathways leading to the onset of oxidative stress, those initiated by the superoxide anions play a central role. This can be explained on the basis that this species is the product of the monoelectronic reduction of dioxygen, which occurs with relative ease under normal conditions in the mitochondria as a consequence of cellular respiration,^18,19^ but also in protein aggregates such as β-amyloid plaques in Alzheimer’s disease as a result of the redox activity of the transition metals embedded in such aggregates.

This scenario led to the testing of the benefit of administering the SOD enzymes themselves to patients. For example, bovine liver Cu,Zn-SOD was first used under the name Orgotein in the late 1970s as an anti-inflammatory agent. The use of Mn-SOD was also investigated, although in this case mainly in gene therapy.^20^ However, it was soon found that the therapeutic administration of SOD enzymes has several drawbacks that prevent their use, such as limited cell permeability, short circulating half-lives, high production cost, and immunogenicity, especially in the case of SODs of nonhuman origin.^20,21^ This has fostered the development of complexes capable of mimicking the activity of SOD enzymes, as a potential way to prevent oxidative stress. Thus, for some time now, different families of small compounds have been studied whose Cu^2+^,^22–29^ Fe^2+^,^22^ Mn^2+^,^20,22,25,30–34^ and quinol-Zn^2+^ ^35^ complexes perform as SOD mimetics. However, as effective as these mimetics are, the very nature of their activity prevents them from eliminating oxidative stress by themselves: hydrogen peroxide is generated as a byproduct of the dismutation of the superoxide radical anion, which is, in turn, a new oxidant species. This is why SOD mimetics can be considered as an antioxidant defense only when coupled to a H_2_O_2_-removing system or when accumulation of this new oxidant does not reach harmful levels.^36^ Indeed, there are several diseases in which the SOD–CAT synergic system fails. In cancer, for instance, the up-regulation of SOD enzymes to control oxidative stress, thus, to avoid cell proliferation, seems not to be coupled with up-regulation of levels of peroxide-removing enzymes, resulting in turn in increased peroxide levels.^37,38^ Although the generation of H_2_O_2_ contributes to the anticancer activity, the accumulated H_2_O_2_ is also used by cancer cells for their proliferation.^36^ It is thus becoming evident that the development of an antioxidant system must be based on a combination of scavengers capable of suppressing both the superoxide radical and hydrogen peroxide.

Different groups have worked on combining the SOD and H_2_O_2_ scavenging activities in mimetic complexes.^39,40^ This has been achieved mainly using Mn^3+^–salen complexes, although other systems based on porphyrins and cyclic polyamines have also been studied.^41,42^ EUK134 is, within the Mn^3+^–salen complexes that constitute the Eukarion family, one of the compounds displaying the best properties from the catalytic activity and the cytotoxicity points of view.^43–46^ However, although these mimetics have better bioavailability and catalytic behavior than administered antioxidant enzymes, having in some cases reached advanced stages of clinical trials,^47^ they also present certain drawbacks.^48^ In addition to the loss of stability due to facile oxidation, a key challenge in optimizing their ROS-scavenging activity is that each catalytic center must mediate both superoxide and hydrogen peroxide decomposition. Thus, the complex will initially catalyze the fastest of both disproportionations. In the more reasonable scenario of faster disproportionation of superoxides, it will lead to a transient increase of the H_2_O_2_ concentration, until the peroxide level increases at levels that make H_2_O_2_ disproportionation competitive with that of O_2_^•–^. During the transient period, the oxidative damage associated with hydrogen peroxide will increase.

To overcome this limitation and to extend the possible approaches, we designed a scaffold capable of coordinating different metals to give rise to mimetics with high, metal-dependent SOD and CAT activities. Specifically, the chosen ligand is based on an azamacrocycle whose Cu^2+^ complexes exhibit some of the highest SOD catalytic constants reported so far (^**COOH**^ **PyNH**_**3**_, see Figure 1).^29^ The ligand has been redesigned by methylation of the secondary amino groups to enhance the SOD activity of its Cu^2+^ complexes^23,49^ and to allow the coordination of Fe^2+^, ensuring the stability of the formed complexes along the catalytic cycle without formation of imine complexes.^50^ Moreover, the carboxylic group of the ligand has been esterified to avoid the potential formation of side species such as coordination polymers in which the carboxylic group of one complex coordinates the Cu^2+^ atom of the next complex. We then studied the speciation and electrochemistry of these ligands with Cu^2+^ and Fe^2+^, as well as the SOD and catalase activities of the resulting complexes.

**Figure 1:**
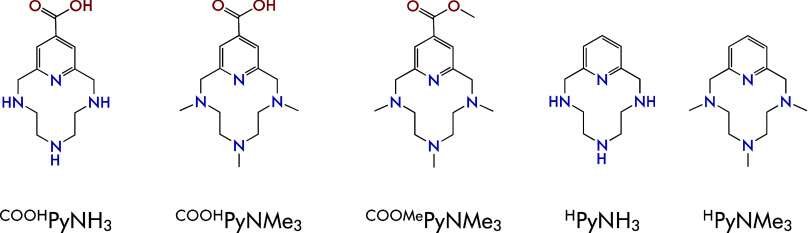
Azamacrocycles studied in this work and analogues reported in the bibliography (^**H**^ **PyNH**_**3**_ and ^**H**^ **PyNMe**_**3**_).^23^

## Results and Discussion

### Acid–Base Behavior

The knowledge of the acid–base behavior of polyamine-type ligands is crucial for understanding their coordination behavior with metal ions. For this reason, we have used potentiometric and UV–vis spectroscopy titrations not only to determine the values of the protonation constants of ^**COOH**^ **PyNMe**_**3**_ and ^**COOMe**^ **PyNMe**_**3**_ but also to get information about their protonation sequence. The protonation constants are shown in Table 1, along with those previously determined for ^**COOH**^ **PyNH**_**3**_.^29^ The representation of the distribution diagrams as a function of the pH can be found in Figures 2, S2, and S3 in the Supporting Information; the spectra of the UV–vis titrations can be found in Figures S4 and S5 in the SI.

**Figure 2:**
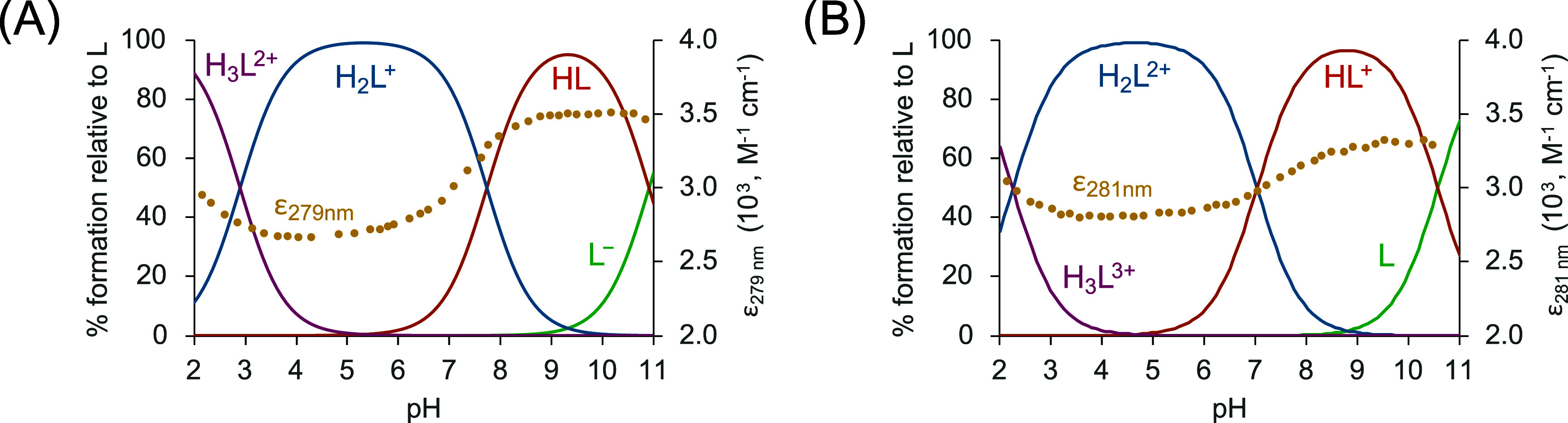
Distribution diagrams of (A) ^**COOH**^ **PyNMe**_**3**_ and (B) ^**COOMe**^ **PyNMe**_**3**_ as a function of the pH in the aqueous solution (0.15 M NaClO_4_, 298.1 ± 0.1 K). The absorbance at ca. 280 nm in the UV–vis spectra is represented overlaid as yellow dots (yellow circle solid).

**Table 1:** Logarithms of the Stepwise Protonation Constants for ^**COOH**^ **PyNH**_**3**_, ^**COOH**^ **PyNMe**_**3**_, and ^**COOMe**^ **PyNMe**_**3**_ Obtained by Potentiometry and UV–Vis Spectroscopy Studies^29^ ^,^ ^a^

reaction^b^	^**COOH**^ **PyNH**_**3**_ ^c^	^**COOH**^ **PyNMe**_**3**_	^**COOMe**^ **PyNMe**_**3**_
$L^{x}+H^{+}⇄{\mathrm{HL}}^{\left(1+x\right)+}$	10.80(1)^d^	10.90(2)	10.57(2)
${\mathrm{HL}}^{\left(1+x\right)+}+H^{+}⇄H_{2}L^{\left(2+x\right)+}$	7.90(1)	7.73(3)	7.03(1)
$H_{2}L^{\left(2+x\right)+}+H^{+}⇄H_{3}L^{\left(3+x\right)+}$	2.25(2)	2.90(4)	2.48(1)^f^
log⁡β ^e^	20.95	21.53	20.18

a The constants were determined in 0.15 M NaClO_4_ at 298.1 ± 0.1 K.

b For ^**COOH**^ **PyNH**_**3**_ and ^**COOH**^ **PyNMe**_**3**_, L corresponds to the form of the ligand in which the carboxylate is deprotonated. *x* takes a value of −1 for ^**COOH**^ **PyNH**_**3**_ and ^**COOH**^ **PyNMe**_**3**_ and 0 for ^**COOMe**^ **PyNMe**_**3**_.

c Values taken from ref 29.

d Values in parentheses are standard deviations in the last significant figure.

e Log β = ∑log *K*.

f Obtained by UV–vis spectroscopic titration.

The potentiometric titrations allowed us to determine three protonation constants both for ^**COOH**^ **PyNMe**_**3**_ and for ^**COOMe**^ **PyNMe**_**3**_, ranging from 10.90 to 2.90 and from 10.57 to 2.48 logarithmic units, respectively (see Table 1). In the case of ^**COOMe**^ **PyNMe**_**3**_, the third protonation constant was determined via UV spectroscopy. The ligands show the typical protonation behavior usually found for cyclic polyamines, which is based on the electrostatic repulsions between protonated amino groups, the inductive effects of the alkyl and aromatic groups, and statistical effects.^51–55^ The values of the three stepwise protonation constants are close for ^**COOH**^ **PyNH**_**3**_, ^**COOH**^ **PyNMe**_**3**_, and ^**COOMe**^ **PyNMe**_**3**_. Indeed, the first two protonation constants are almost equal for the carboxylate ligands regardless of whether the amino groups of the macrocycle are methylated or not. On the other hand, the carboxylated ligands show slightly higher protonation constants than ^**COOMe**^ **PyNMe**_**3**_ (see Figure S3 in the SI). These values are consistent with those previously reported for similar systems and can be explained, among other factors, by the formation of internal hydrogen bonds within the macrocyclic structures.^23^ This conclusion is also supported by UV spectroscopy studies. When following the absorbance band of the pyridine group at 279 nm with pH, we can observe a significant increase in intensity from pH 4 to 2, as the triprotonated species is formed (according to the distribution diagrams). Since this rise of the absorbance can also be observed in the titration of ^**COOMe**^ **PyNMe**_**3**_, it can be interpreted by assuming that the third protonation implies that the last amine of the macrocycle is protonated in place of the pyridine carboxylate.

### Interaction with Cu^2+^ and Fe^2+^ Ions

As a preliminary step in the analysis of the antioxidant activity of the Cu^2+^ and Fe^2+^ complexes, the interaction of such metal ions with ^**COOH**^ **PyNMe**_**3**_ and ^**COOMe**^ **PyNMe**_**3**_ was studied by pH-metric titrations and UV–vis spectroscopy. The stability constants obtained, as well as those previously described for ^**COOH**^ **PyNH**_**3**_,^23^ are shown in Table 2. The representation of the distribution diagrams as a function of the pH can be found in Figures 3 and S6. It should be noted that the oxidation state 2+ of iron was ensured and checked thoroughly along the different studies. The Fe^2+^ stock solutions were prepared in the presence of HNO_3_ (0.1 M) to avoid a rapid oxidation to Fe^3+^. This solution was freshly prepared before each assay. Moreover, the absence of Fe^3+^ in the solutions of ^**COOH**^ **PyNMe**_**3**_ or ^**COOMe**^ **PyNMe**_**3**_ with Fe^2+^ was ensured by deaerating them with an argon flow. The absence of Fe^2+^ oxidation was verified by adding xylenol orange and observing the absence of the characteristic absorbance band of the xylenol orange–Fe^3+^ complex at 589 nm (see Figure S9).

**Figure 3:**
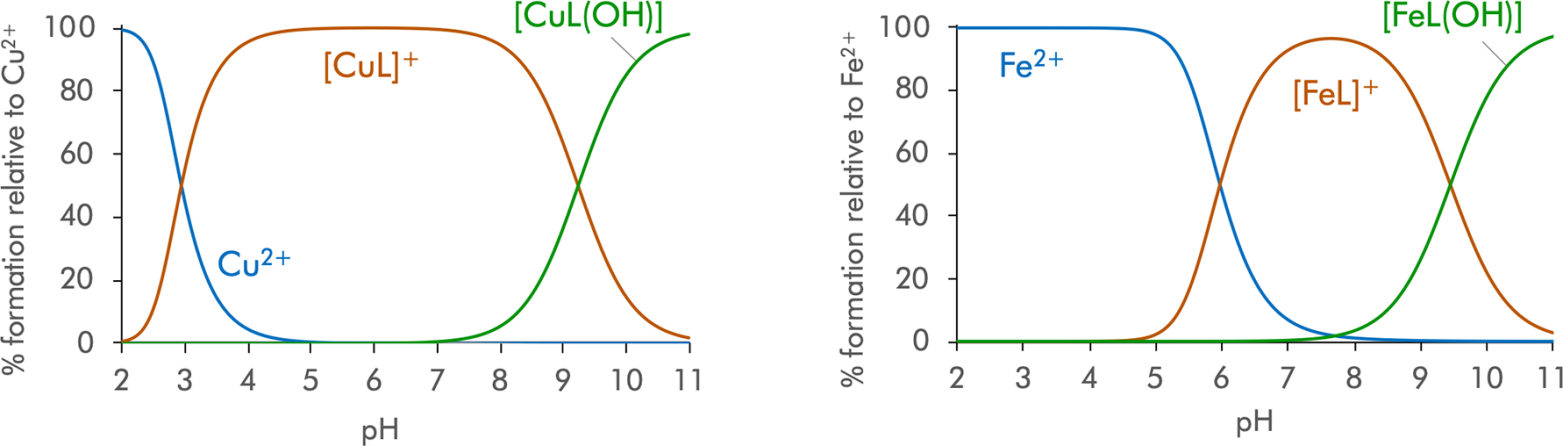
Species distribution curves for the Cu^2+^ (left) and Fe^2+^ (right) complexes in an aqueous solution (0.15 M NaClO_4_, 298.1 ± 0.1 K) of ^**COOH**^ **PyNMe**_**3**_. L corresponds to the form of the ligand in which the carboxylate is deprotonated. [Cu^2+^]_0_ = [Fe^2+^]_0_ = [L]_0_ = 10^–3^ M.

**Table 2:** Logarithms of the Stepwise Stability Constants for the Cu^2+^ and Fe^2+^ Complexes of ^**COOH**^ **PyNH**_**3**_, ^**COOH**^ **PyNMe**_**3**_, and ^**COOMe**^ **PyNMe**_**3**_ Obtained by Potentiometric Measurements^a^

reaction^b^	^**COOH**^ **PyNH**_**3**_	^**COOH**^ **PyNMe**_**3**_	^**COOMe**^ **PyNMe**_**3**_
Cu+L⇄[CuL]	17.62(2)^c^,^d^	16.35(4)	16.84(3)
$\left[\mathrm{CuL}\right]+H_{2}O⇄\left[\mathrm{CuL}\left(\mathrm{OH}\right)\right]+H$	–9.07(2)^c^	–9.24(7)	–8.26(4)
Fe+L⇄[FeL]		10.02(2)	9.46(2)
$\left[\mathrm{FeL}\right]+H_{2}O⇄\left[\mathrm{FeL}\left(\mathrm{OH}\right)\right]+H$		–9.44(4)	–8.18(5)

a The constants were determined in 0.15 M NaClO_4_ at 298.1 ± 0.1 K.

b For ^**COOH**^ **PyNH**_**3**_ and ^**COOH**^ **PyNMe**_**3**_, L corresponds to the form of the ligand in which the carboxylate is deprotonated. Charges have been omitted for simplicity.

c Values taken from ref 29.

d Values in parentheses are standard deviations in the last significant figure.

The ligands form stable [CuL] and [CuL­(OH)] complexes in the whole pH range studied. The values of the stability constants of [Cu­(^**COO**^ **PyNMe**_**3**_)]^+^ and [Cu­(^**COOMe**^ **PyNMe**_**3**_)]^2+^ are slightly smaller than that of [Cu­(^**COO**^ **PyNH**_**3**_)]^+^, which can be explained attending to the hydrophobic character of the alkylic substituents and the subsequent poorer solvation of the metal center. This observation agrees with prior results we and others have reported, in which a similar decrease in the stability of the complexes is associated with the poorer σ-donor ability (related to the reduction in the number of M–N–H···O hydrogen bonds with the water solvent molecules) and the increase of the *radii* of the complexes.^23,56,57^ The prevalent species at physiological pH is [CuL] for the three ligands, with 98.0% of formation for [Cu­(^**COO**^ **PyNH**_**3**_)]^+^, 98.6% for [Cu­(^**COO**^ **PyNMe**_**3**_)]^+^, and 87.9% for [Cu­(^**COOMe**^ **PyNMe**_**3**_)]^2+^.

On the other hand, the speciation studies of the Fe^2+^ complexes (Table 2, Figures 3 and S6 in the SI) show the formation of two mononuclear species: [FeL] and its monohydroxilated form [FeL­(OH)]. Although the Fe^2+^ stability constants are lower than those of the Cu^2+^ systems, the formation of the Fe^2+^ complexes presents a behavior similar to that observed for the Cu^2+^ complexes. The smaller value of these stability constants can be explained by the influence of the radius of the metal ions,^58^ the crystal field stabilization, and the Jahn–Teller effect. The prevalent species at physiological pH is [FeL], with 96.1% for [Fe­(^**COO**^ **PyNMe**_**3**_)]^+^, and 84.0% for [Fe­(^**COOMe**^ **PyNMe**_**3**_)]^2+^, in solutions containing equimolar concentrations of L and Cu^2+^ or Fe^2+^ at 10^–3^ M. Finally, it should be noted that a slow hydrolysis of the ester group in ^**COOMe**^ **PyNMe**_**3**_ has been observed for basic solutions. This slightly increases the uncertainty for the behavior of this ligand and the corresponding complexes at pH higher than 9. Neither mixed Cu^2+^–Fe^2+^ nor other bimetallic complexes have been identified. Further analysis of the ligand’s interaction with Cu^2+^ and Fe^2+^ ions can be found in the Supporting Information.

The formation of complexes in solution was confirmed by the UV–vis spectra recorded for solutions containing equimolar amounts of the ligands and the Cu^2+^ (Figure S7) or Fe^2+^ (Figure S8) metal ions, which show bands at ca. 690–705 nm (Cu^2+^) and ca. 420 nm (Fe^2+^) similar to those observed for complexes with related macrocyclic polyamines.^23,49,50^ The cyclic voltammograms (CVs) in the next section add further support to the formation of M^2+^–L complexes.

### Electrochemistry in Aqueous Solution

Figure 4 shows the superimposed cyclic voltammograms (CVs) at a glassy carbon electrode (GCE) for a 10^–3^ M ^**COOH**^ **PyNMe**_**3**_ plus 5 × 10^–4^ M Cu^2+^ solution in 50 mM TRIS buffer at pH 7.4, with the potential scan reversed at different potentials. Excess ligand was in part used to ensure full complexation of the metal ion. The voltammograms are dominated by a prominent cathodic wave at −0.60 V preceded by an ill-defined shoulder at −0.5 V and followed, in the subsequent anodic scan, by a tall peak at −0.05 V, which is accompanied by a weak anodic signal at ca. +0.35 V. The anodic peak at −0.05 V corresponds to a typical copper stripping and denotes that the reductive process at −0.60 V results in the formation of the Cu metal. The stripping peak disappears when the potential is switched at −0.50 V, thus suggesting that at these potentials the Cu^2+^ species are reduced to Cu^+^ ones. In turn, the signal at +0.35 V can be attributed to the formation of copper–halide complexes (the receptor ^**COOH**^ **PyNMe**_**3**_ was prepared as a chloride salt) displaying Cu^2+^/Cu^+^ interconversion at these potentials. This voltammetry can be interpreted in terms of the reduction of the Cu^2+^–^**COOH**^ **PyNMe**_**3**_ complex to metallic copper involving an unstable intermediate Cu^+^ species. In air-saturated solutions, this voltammetry experiences only minor changes, thus denoting that no significant interaction exists with dissolved O_2_ and its reduction products (see Supporting Information, Figure S10). Although there is no direct access to the formal potential of the Cu^2+^–^**COOH**^ **PyNMe**_**3**_ **/**Cu^+^–^**COOH**^ **PyNMe**_**3**_ couple, an estimate can be made from the midpeak potential of the first cathodic signal in Figure 4, around −0.40 V vs Ag/AgCl, corresponding to −0.20 V vs SHE (see Table 3).

**Figure 4:**
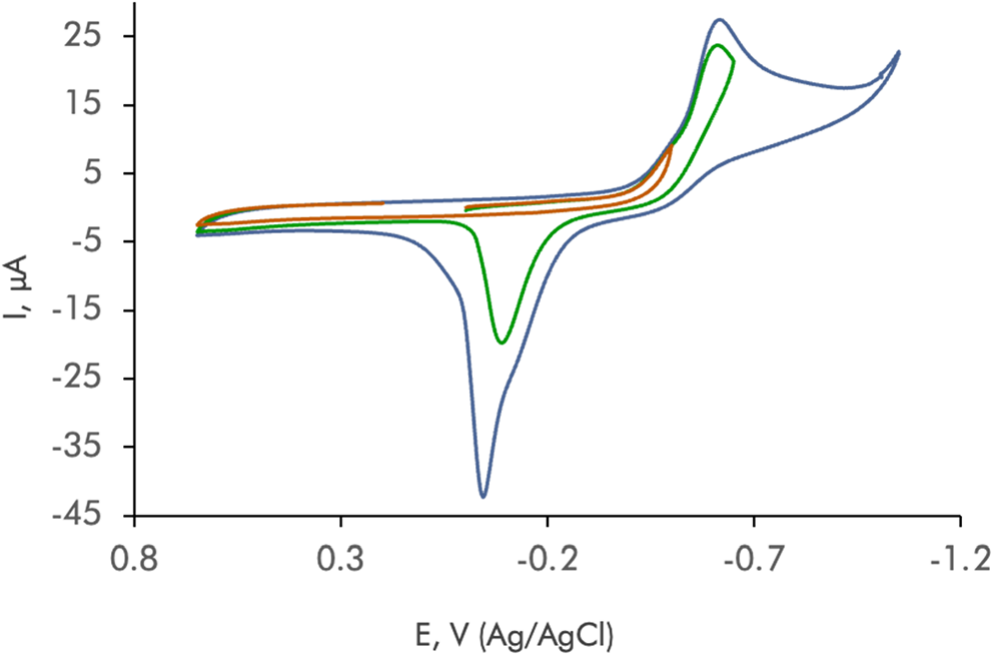
CVs at the GCE of a 10^–3^ M ^**COOH**^ **PyNMe**_**3**_ plus 5 × 10^–4^ M Cu^2+^ solution in 50 mM TRIS buffer at pH 7.4. Potential scan initiated at 0.0 V in the negative direction: potential scan rate, 50 mV s^–1^. Three voltammograms are superimposed corresponding to experiments in which the scan was reversed at different potentials (−1.05 V in blue, −0.65 V in green, and −0.5 V in red).

**Table 3:** Potential for the Reactions Involving the Cu^2+^ or Fe^2+^ Complexes and the Superoxide Anion or Hydrogen Peroxide (Potentials vs SHE at pH 7.4)

entry	reaction	*E*°′ (V)
1	1 Cu2+−PCOOHyNMe3+O2•−→Cu+−PCOOHyNMe3+O2	0.13
2	2 Cu+−PCOOHyNMe3+O2•−+2H+→Cu2+−PCOOHyNMe3+H2O2	1.11
3	3 Cu2+−PCOOHyNMe3+1/2H2O2→Cu+−PCOOHyNMe3+1/2O2+H+	–0.34
4	4 Cu+−PCOOHyNMe3+1/2H2O2+H+→Cu2+−PCOOHyNMe3+1/2H2O	0.86
5	5 Fe3+−PCOOHyNMe3+O2•−→Fe2+−PCOOHyNMe3+O2	0.66
6	6 Fe2+−PCOOHyNMe3+O2•−+2H+→Fe3+−PCOOHyNMe3+H2O2	0.58
7	7 Fe3+−PCOOHyNMe3+1/2H2O2→Fe2+−PCOOHyNMe3+1/2O2+H+	0.19
8	8 Fe2+−PCOOHyNMe3+1/2H2O2+H+→Fe3+−PCOOHyNMe3+1/2H2O	0.33

The voltammetry of Fe^2+^ complexes is dominated by a quasi-reversible one-electron redox couple at 0.13 V vs Ag/AgCl (0.33 V vs SHE) with peak potentials of −0.05 (cathodic) and +0.15 V (anodic) (see Supporting Information, Figure S11). The voltammetric response of solutions containing a mixture of copper and iron complexes reproduces the essential features recorded for the individual solutions (see Supporting Information, Figure S12), thus denoting the absence of mutual interaction under our experimental conditions. Table 3 summarizes the estimated values of the formal potential (*E*°′) of the Cu^2+^–^**COOH**^ **PyNMe**_**3**_ **/**Cu^+^–^**COOH**^ **PyNMe**_**3**_ and Fe^3+^–^**COOH**^ **PyNMe**_**3**_ **/**Fe^2+^–^**COOH**^ **PyNMe**_**3**_ couples. From these values of potentials and those reported for the different pairs involving O_2_, O_2_^–^, and H_2_O_2_, it can be deduced that the processes in entries 1–2 of Table 3 are thermodynamically favored, in agreement with the observed SOD activity of the Cu^2+^–^**COOH**^ **PyNMe**_**3**_ complex. In contrast, the potential for entry 3 indicates that no catalase activity should be expected for the Cu^2+^ complex, which is also in agreement with the experimental observation. The observed catalase activity of Fe^2+^–^**COOH**^ **PyNMe**_**3**_ can also be justified by the potentials for entries 7–8, but the values for entries 5–6 indicate that this complex should also catalyze superoxide disproportionation. The observed lack of SOD activity may be because after oxidation to Fe^3+^–^**COOH**^ **PyNMe**_**3**_, there is no reduction to the Fe^2+^ species but subsequent oxidation to higher valent species that do not result in O_2_ release, at least under the present experimental conditions.

### Superoxide Dismutase Activity

Once characterized the Cu^2+^ and Fe^2+^ coordination chemistry in solution, we carried out antioxidant activity assays. At this point, we focused on the ability of the studied systems to remove two of the main ROS: the superoxide radical and hydrogen peroxide.

First, the SOD activity of the complexes was studied by means of the nitroblue tetrazolium (NBT) method, also known as McCord–Fridovich method.^59^ This is a widely used, indirect method based on the side production of superoxide anions by the xanthine oxidase enzyme and their capture by the NBT dye to give formazan.^60–63^ A compound displaying SOD activity decreases the flow of superoxide radical anions and, thereby, the production of formazan (see the representation of the fitting of the data in Figures 5 and S13–S16). Thus, by following the absorption band of formazan at 560 nm, the SOD activity of the studied mimetic can be quantified. Blank experiments were recorded with the ligands alone without observing any effect. The values of IC_50_, defined as the amount of the compound necessary for achieving 50% inhibition of NBT reduction to formazan by the superoxide anion, together with the catalytic constants (*k*_cat_), were obtained as indicated in the Experimental Section and are summarized in Table 4.

**Figure 5:**
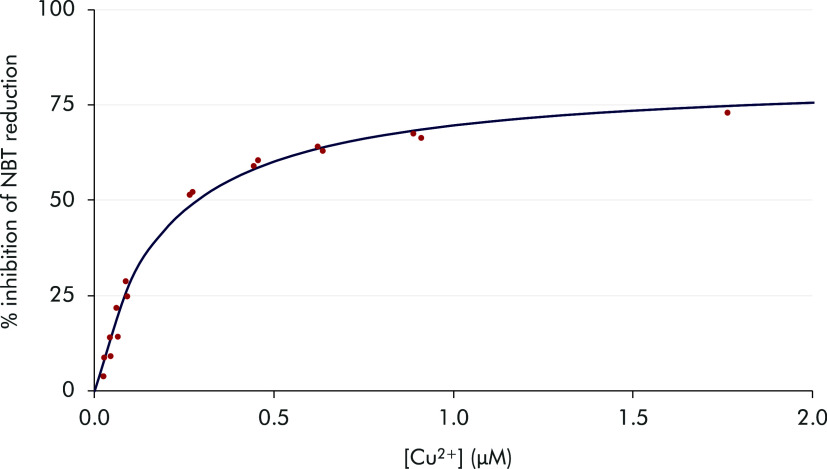
Fitting of the SOD activity data obtained by the McCord–Fridovich method for the system Cu^2+^–^**COOH**^ **PyNMe**_**3**_.

**Table 4:** Evaluation of the SOD Activity of the Cu^2+^ and Fe^2+^ Complexes with the Studied Ligands in 50 mM TRIS Buffer at pH 7.4^a^

system	IC_50_ (μM)	*k*_cat_ (10^6^ M^–1^ s^–1^)
Cu-^**COOH**^ **PyNH**_**3**_ ^c^	0.36(1)^b^	9.7
Cu-^**COOH**^ **PyNMe**_**3**_	0.29(3)	12.3
Cu-^**COOMe**^ **PyNMe**_**3**_	0.09(1)	40.3
Cu-^**H**^ **PyNH**_**3**_ ^d^	2.1(4)	1.7
Cu-^**H**^ **PyNMe**_**3**_ ^d^	2.9(6)	1.2
Cu-^**NMe2**^ **PyNH**_**3**_ ^e^	1.69	1.6
Cu-^**CN**^ **PyNH**_**3**_ ^e^	0.70	3.8
Cu-^**CF3**^ **PyNH**_**3**_ ^e^	0.13	20.1
all Fe^II^ complexes	>100	<0.01
Cu(ClO_4_)_2_ ^f^	1.1(1)	2.7
Cu_2_Zn_2_–SOD^f^	0.010(2)	430

a Some literature data are also included for comparison. [NBT] = 59 μM.

b Values in parentheses are standard deviations in the last significant figure.

c Reference 29.

d Reference 23.

e Reference 28.

f Reference 64.

The results show that the SOD activity is strongly dependent on the nature of the metal ion. Although the iron complexes are inactive, both the IC_50_ and the *k*_cat_ values show that the activity of the Cu^2+^ complexes is very remarkable, ranking among the best mimetics so far reported in the bibliography (see Figure 6).^23,25–29^ In particular, the *k*_cat_ value for Cu^2+^–^**COOMe**^ **PyNMe**_**3**_ only differs by about 1 order of magnitude from that of CuZn-SOD.^23^ The introduction of both the *p*-COOH and *p*-COOMe groups in ^**H**^ **PyNH**_**3**_ or ^**H**^ **PyNMe**_**3**_ leads to a significant enhancement of the SOD activity of the Cu^2+^ complexes. Indeed, at the same concentration at which the complexes of ^**H**^ **PyNH**_**3**_ and ^**H**^ **PyNMe**_**3**_ inhibit 50% of the NBT reduction, the carbonylic complexes have already reached the *plateau* of activity, displaying inhibition levels of 80% (see Figures S13–S16 in the Supporting Information). As for the catalytic constants, functionalization of the ligands with a COOH group leads to a 6- to 10-fold increase in the catalytic constant for both the ^**COOH**^ **PyNH**_**3**_ and ^**COOH**^ **PyNMe**_**3**_ complexes (see Figure S17).^23^ What is even more noteworthy is the fact that when the pyridine is functionalized with a methyl ester group (^**COOMe**^ **PyNMe**_**3**_) instead of with a carboxylic acid (^**COOH**^ **PyNMe**_**3**_), the enhancement rises 34-fold (Table 4 and Figure S17). This behavior could be explained by the more electron-withdrawing character of the functionalized carboxylic pyridine.

**Figure 6:**
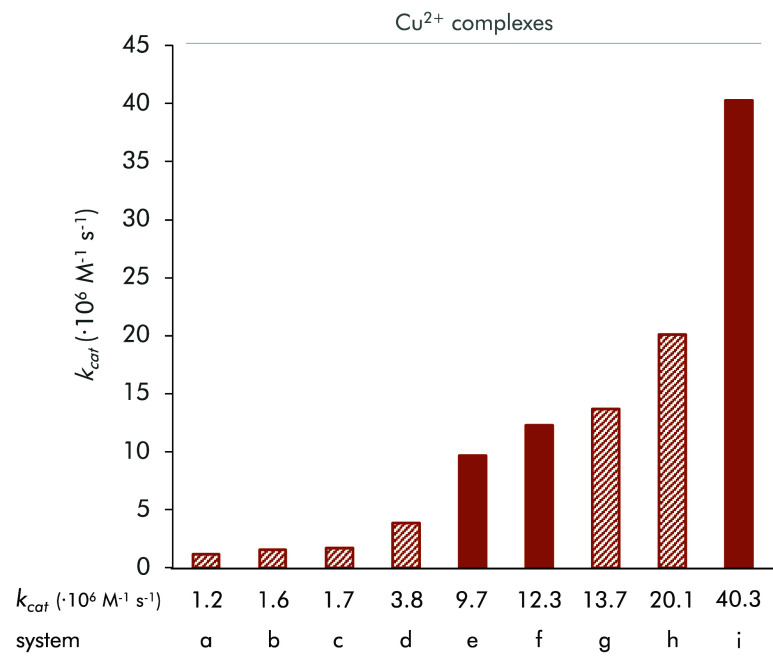
Representation of the catalytic constant values of the systems (a) Cu^2+^-^**H**^ **PyNMe**_**3**_,^23^ (b) Cu^2+^-^**NMe2**^ **PyNH**_**3**_,^28^ (c) Cu^2+^-^**H**^ **PyNH**_**3**_,^23^ (d) Cu^2+^-^**CN**^ **PyNH**_**3**_,^28^ (e) Cu^2+^-^**COOH**^ **PyNH**_**3**_, (f) Cu^2+^-^**COOH**^ **PyNMe**_**3**_, (g) Cu^2+^-^**H**^ **PyN­(** ***i-*** **Pr)**_**2**_ **Me**,^23^ (h) Cu^2+^-^**CF3**^ **PyNH**_**3**_,^28^ and (i) Cu^2+^-^**COOMe**^ **PyNMe**_**3**_.

Unlike the Cu^2+^ complexes, the Fe^2+^ complexes of ^**COOH**^ **PyNH**_**3**_, ^**COOH**^ **PyNMe**_**3**_, and ^**COOMe**^ **PyNMe**_**3**_ show negligible SOD activity under the conditions of this study, which makes this ion uncompetitive with Cu^2+^ for superoxide disproportionation.

### Catalase Activity

The ability of the complexes for removing H_2_O_2_, thus mimicking the activity of the catalase enzymes, was checked by monitoring the pressure changes at 25 °C during the reaction of H_2_O_2_ with the ^**COOH**^ **PyNMe**_**3**_ complexes of Fe^2+^ and Cu^2+^ in aqueous solutions buffered at pH 7.5 (see speciation curves in Figures S18 and S19). The results indicate that the Fe^2+^ complex carries out H_2_O_2_ decomposition under catalytic conditions, whereas the Cu^2+^ complex is inactive. UV–vis monitoring of the reaction of the Cu^2+^ complex with H_2_O_2_ did not show significant spectral changes, in agreement with its lack of catalase activity.

The catalase activity of the Fe^2+^–^**COOH**^ **PyNMe**_**3**_ complex was evaluated at different concentrations of the complex and oxidant (see speciation curves in Figures S18 and S19), and the experimental curves are shown in Figures 7 and S20. Those curves can be easily transformed to derive the moles of O_2_ formed in the gas phase (Figure S21) by using the ideal gas equation, the experimental concentrations, and the reactor volume. The total amount of O_2_ and the corresponding values of TON for each experiment (Table 5), calculated as the number of moles of H_2_O_2_ decomposed by one mol of the metal ion, show that the Fe^2+^–^**COOH**^ **PyNMe**_**3**_ complex presents a significant catalase activity while the Cu^2+^–^**COOH**^ **PyNMe**_**3**_ complex does not display any significant pressure change after several hours of reaction. The present results for the iron complex compare well with those reported for the Mn^2+^ complexes of ^**H**^ **PyNH**_**3**_, ^**OH**^ **PyNH**_**3**_, and ^**MeO**^ **PyNH**_**3**_, which have been recently reported to show catalase activity with TON (25–37) values close to those of Fe^2+^–^**COOH**^ **PyNMe**_**3**_.^65^

**Figure 7:**
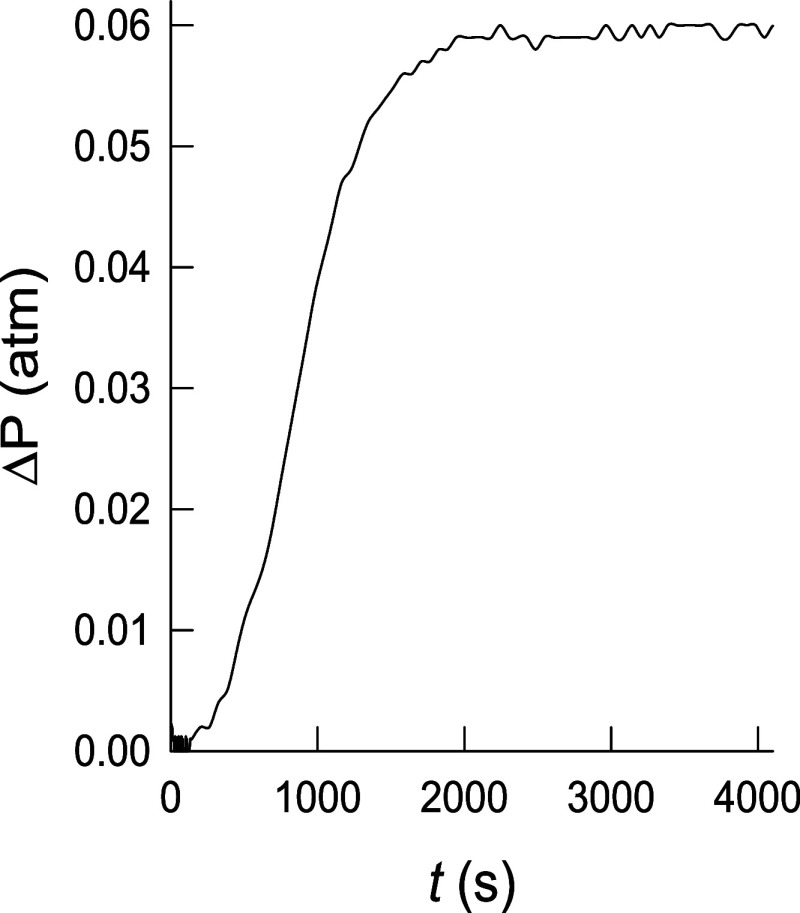
Typical pressure changes during the reaction of the Fe^2+^–^**COOH**^ **PyNMe**_**3**_ complex with H_2_O_2_ at pH 7.5 and 25 °C ([^**COOH**^ **PyNMe**_**3**_]_0_ = 2.14 × 10^–4^ M, [Fe^2+^]_0_ = 1.93 × 10^–4^ M, [H_2_O_2_]_0_ = 3.4 × 10^–2^ M).

**Table 5:** Catalase Activity of the Fe^2+^–**^COOH^PyNMe**_**3**_ Complex Evaluated from Pressure Monitoring Experiments at 25°C and pH 7.5^a^

entry	[**^COOH^ **P**yNMe**_**3**_]_0_	[Fe^2+^]_0_ (M)^b^	[Cu^2+^]_0_ (M)^c^	[H_2_O_2_]_0_ (M)	n_O2_(g) (moles)	TON^d^
1	2.73 × 10^–4^	2.46 × 10^–4^		1.1 × 10^–2^	7.26 × 10^–6^	17
2	2.5 × 10^–4^	2.25 × 10^–4^		2.0 × 10^–2^	1.33 × 10^–5^	35
3	2.14 × 10^–4^	1.93 × 10^–4^		3.4 × 10^–2^	2.61 × 10^–5^	81
4	1.07 × 10^–4^	9.63 × 10^–5^		3.4 × 10^–2^	2.04 × 10^–5^	126
5	5.36 × 10^–5^	4.82 × 10^–5^		3.4 × 10^–2^	1.56 × 10^–5^	193
6	2.14 × 10^–4^		1.93 × 10^–4^	3.4 × 10^–2^	0	0
7	2.14 × 10^–4^	8.57 × 10^–5^	8.57 × 10^–5^	3.4 × 10^–2^	1.18 × 10^–5^	41^f^
8		2.14 × 10^–4^		3.4 × 10^–2^	0	0
9		2.14 × 10^–4^ ^e^		3.4 × 10^–2^	0	0
10			2.7 × 10^–4^	4.3 × 10^–2^	0	0
11				3.4 × 10^–2^	0	0
12	2.14 × 10^–4^			3.4 × 10^–2^	0	0

a The results obtained for iron and copper salts, as well as for an equimolar mixture of both metal ions, are also included.

b Fe­(SO_4_).

c Cu­(ClO_4_)_2_.

d Calculated as the number of moles of H_2_O_2_ decomposed by one mol of metal ions.

e FeCl_3_.

f Calculated for the total amount of metal ions (Cu^2+^ + Fe^2+^). The TON value considering only Fe^2+^ would be 82, in good agreement with entry 3.

Regarding the nature of the species in solution under the conditions of the experiments, the speciation curves calculated from the stability constants (Figures S18 and S19) indicate that the major species in solution under the conditions of the pressure monitoring experiments is [FeL] (ca. 88–94%). [FeL­(OH)] only reaches ca. 1% of formation, but the concentration of free Fe^2+^ represents around 4.8–10.9% of the total iron. Thus, there is the possibility that the catalase activity observed is due, totally or in part, to the activity of free iron. To clarify this point, pressure monitoring experiments using similar concentrations of Fe^2+^ and Fe^3+^ salts in the absence of ligands were carried out, and no gas evolution was observed. This finding agrees with the slow kinetics of decomposition of H_2_O_2_ at neutral pH observed for free Fe^2+^ and Fe^3+^ ^66^ and indicates that the activity observed in the presence of the ligand is exclusively caused by the Fe^2+^–^**COOH**^ **PyNMe**_**3**_ complex. Entry 7 in Table 5 includes the results for an equimolar mixture of Fe^2+^ and Cu^2+^, which indicate that the catalase activity of the Fe^2+^ complex is preserved in the presence of the inactive Cu^2+^ complex.

## Conclusions

The results presented in this work indicate that the complexes formed by newly prepared ^**COOH**^ **PyNMe**_**3**_ and ^**COOMe**^ **PyNMe**_**3**_ macrocycles have promising antioxidant properties *in vitro*, making them attractive scaffolds for the treatment of ROS-related diseases. Of relevance is their capability to disproportionate O_2_^•–^ or H_2_O_2_ in a metal-selective manner, Cu^2+^ being only active toward superoxide dismutation and Fe^2+^ only toward hydrogen peroxide disproportionation. This opens the possibility to adjust the ratio of SOD and CAT activity via the amount of Fe or Cu bound, which could then be adapted to the need in a specific biological site. The activity changes observed when the metal ion is changed cannot be explained with simple considerations based on the redox potentials, thus indicating a more complex mechanism. Further work is required to check the validity of this strategy for developing new antioxidant drugs for the treatment of diseases associated with oxidative stress. In addition to *in vivo* experiments, experimental work is also required to obtain detailed information about the reactivity and reaction mechanisms of the complexes toward the O_2_^•–^ and H_2_O_2_ oxidants. Although the reasons leading to the very different and highly metal-dependent selective performance toward these oxidants only could be speculative with the current information, the great performance shown by the analyzed enzyme mimetics encourages their further study.

## Experimental Section

### Synthesis of the Ligands

^**COOH**^ **PyNH**_**3**_ was synthesized following the procedure previously described, as outlined in Scheme 1.^29^ ^**COOH**^ **PyNH**_**3**_ was methylated by means of a slight variation of the Eschweiler–Clarke reaction, yielding ^**COOH**^ **PyNMe**_**3**_ in >98% yield.^49,50^ Finally, the carboxylic group of ^**COOH**^ **PyNMe**_**3**_ was esterified under acidic conditions to yield ^**COOMe**^ **PyNMe**_**3**_·3HCl in >98%. All reagents were obtained from commercial sources and used as received. Solvents used for the chemical synthesis were of analytical grade and used without further purification. Characterization of the final compounds can be found in the Supporting Information file (Figures S22–S30).

**Scheme 1:**
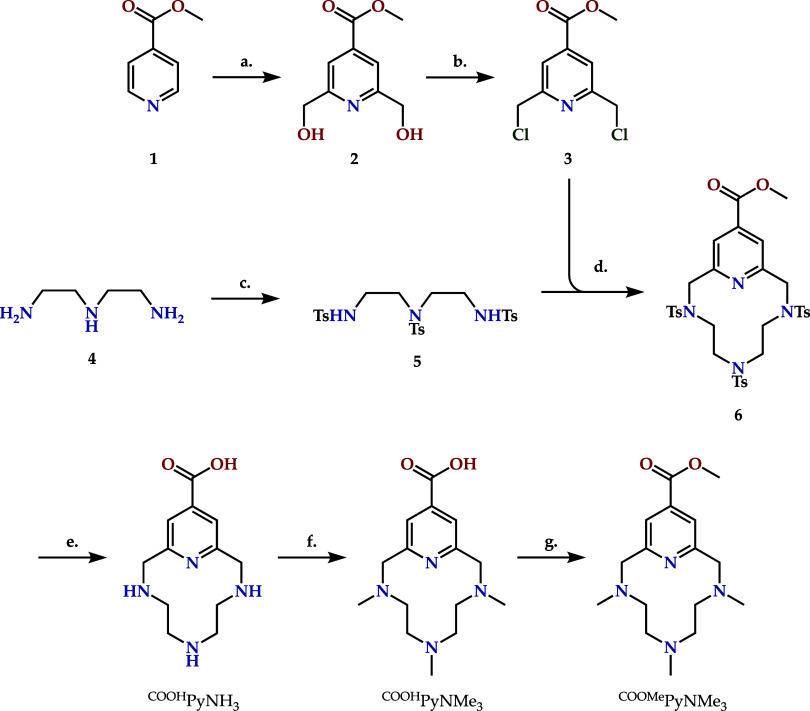
Synthetic Route for the Preparation of the Polyaza-macrocyclic Receptor **L**, from Methyl Isonicotinate and Diethylenetriamine[Fn s1fn1]

a
*Reagents and conditions:* (a) (NH_4_)_2_S_2_O_8_, FeCl_2_, H_2_SO_4_, MeOH, H_2_O, reflux for 36 h, 58%; (b) MsCl, Et_3_N, CH_2_Cl_2_, reflux for 12 h, 46%; (c) TsCl, K_2_CO_3_, THF, H_2_O, r.t. for 24 h, 93%; (d) K_2_CO_3_, CH_3_CN_anh._, reflux for 24 h, 94%; (e) H_2_SO_4_, 110 °C for 5 h, 71%; (f) HCOOH, CH_2_O, H_2_O, 100 °C for 5 days, >98%; and (g) HCl, MeOH, reflux for 72 h, >98%.

#### Synthesis of ^**COOH**^ **PyNMe**_**3**_·3HCl

The deprotected macrocycle ^**COOH**^ **PyNH**_**3**_ (0.30 g, 0.57 mmol), formic acid (1.01 mL, 24.33 mmol), and formaldehyde (0.73 mL, 26.31 mmol) were dissolved in 5 mL of Milli-Q water and stirred at 100 °C for 5 days.^49^ Then, the solvent and both liquid reagents (the acid and aldehyde) were removed under reduced pressure. Once dryness was reached, the salt of the methylated pure compound was obtained as a pale brown solid (0.23 g, >98%). Characterization data of the compound are included in the Supporting Information (Figures S25–S27). ^1^H NMR (300.1 MHz, D_2_O), δ (ppm): 7.97 (s, 2H), 4.82 (s, 4H), 3.52 (t, *J* = 5.5 Hz, 4H), 3.21 (s, 6H), 2.69–2.40 (m, 4H), 2.47 (s, 3H). ^13^C NMR (75.5 MHz, D_2_O), δ (ppm): 150.7, 121.9, 60.8, 56.0, 51.9, 44.4, 41.1. MS *m*/*z* (ESI) = 293.1 g mol^–1^ ([M + H]^+^).

#### Synthesis of ^**COOMe**^ **PyNMe**_**3**_·3HCl

^**COOH**^ **PyNMe**_**3**_·3HCl (0.33 g, 0.57 mmol) and HCl (50 μL) were dissolved in MeOH (50 mL). The resulting mixture was refluxed for 72 h, and after cooling down, the solvent was evaporated to dryness under reduced pressure. The pale brown solid was dissolved in water and basified with Na_2_CO_3_ (5 mL, pH ca. 9–10), the resulting aqueous solution was extracted with CH_2_Cl_2_ (3 × 10 mL), and the combined organic phases were dried with anhydrous Na_2_SO_4_. After filtering off the solid, 2 mL of HCl/dioxane was added to the solution, thus precipitating the compound ^**COOMe**^ **PyNMe**_**3**_ as a hydrochloride salt. The suspension was centrifuged, and the solvent was finally removed. The compound ^**COOMe**^ **PyNMe**_**3**_ was obtained as a pale brown solid in the hydrochloride salt form (0.24 g, >98%). Characterization data of the compound are included in the Supporting Information (Figures S28–S30). ^1^H NMR (300.1 MHz, D_2_O) δ, ppm: 8.01 (s, 2H), 4.82 (s, 4H), 3.99 (s, 3H), 3.49 (t, *J* = 5.5 Hz, 4H), 3.21 (s, 6H), 2.73–2.47 (m, 4H), 2.42 (s, 3H). ^13^C NMR (75.5 MHz, D_2_O) δ, ppm: 166.0, 150.7, 140.9, 121.8, 60.8, 56.2, 53.5, 52.0, 44.4, 41.0. ESI-MS, *m*/*z*: [M + H]^+^ calculated 307.21, found 307.1. Anal. Calcd for C_16_H_26_O_2_N_4_·3HCl: C, 46.22; H, 7.03; N, 13.47: Found: C, 46.19; H, 7.21; N, 13.5.

### NMR Measurements

The ^1^H and ^13^C NMR spectra were recorded on a Bruker Advance DPX300 spectrometer operating at 300.13 MHz for ^1^H and 75.47 MHz for ^13^C. For the ^1^H spectra, the solvent signal was used as a reference standard.

### Preparation of the Stock Solutions

Stock solutions of Cu^2+^, HNO_3_, Fe^2+^, TRIS, HEPES, ^**COOH**^ **PyNH**_**3**_, ^**COOH**^ **PyNMe**_**3**_, and ^**COOMe**^ **PyNMe**_**3**_ were prepared. Stock solutions of Cu^2+^ of ca. 0.1 M were prepared in Milli-Q water from the CuCl_2_·2H_2_O salt. The solutions were standardized through a direct quantification with UV–vis spectroscopy (ε_810_ = 12 M^–1^ cm^–1^) and by means of a titration with EDTA (ε_730_ = 87 M^–1^ cm^–1^). A 0.1 M solution of HNO_3_ was prepared by dilution of commercial nitric acid (65% w/w) purchased from Scharlau in Milli-Q water. Fe^2+^ stock solutions of ca. 10^–3^ M were prepared by dissolving FeSO_4_·7H_2_O in the 0.1 M solution of HNO_3_ to avoid a rapid oxidation of Fe^2+^ to Fe^3+^. This solution was freshly prepared before each assay, and the absence of ferric iron was checked by spectroscopic measurements. Two stock solutions of TRIS and HEPES buffers (50 mM) were prepared by a solution of the free acids in Milli-Q water. The pH was adjusted with NaOH and HCl to 7.4 in both cases. The stock solutions of the ligands (^**COOH**^ **PyNH**_**3**_, ^**COOH**^ **PyNMe**_**3**_, and ^**COOMe**^ **PyNMe**_**3**_; 10 mM) were made through solution of the hydrochloric salts of each compound in the TRIS buffer (50 mM, pH 7.4). The absence of Fe^3+^ in the solutions of ^**COOH**^ **PyNMe**_**3**_ or ^**COOMe**^ **PyNMe**_**3**_ with Fe^2+^ was ensured by deaerating them with an argon flow. The null oxidation of Fe^2+^ was checked by the addition of xylenol orange and the subsequent absence of the characteristic absorbance band of the xylenol orange–Fe^3+^ complex at 589 nm (see Figure S9).

### Potentiometric Studies

*Caution! Perchlorate salts are explosive and should be handled with care; such compounds should never be heated as solids*. The potentiometric titrations were carried out at 298.1 ± 0.1 K using 0.15 M NaClO_4_ as the supporting electrolyte. The experimental procedure (buret, potentiometer, cell, stirrer, microcomputer, etc.) has been fully described elsewhere.^67^ The acquisition of raw potentiometric data was performed with the computer program PASAT.^68^ The reference electrode was a Ag/AgCl electrode in a saturated KCl solution. The glass electrode was calibrated as a hydrogen-ion concentration probe by titration of previously standardized amounts of HCl with CO_2_-free NaOH solutions, and the equivalent point was determined by Gran’s method,^69,70^ which gives the standard potential, E̊, and the ionic product of water (p*K*_w_ = 13.73(1)). The computer program HYPERQUAD was used to calculate the protonation and stability constants.^71^ The HYSS^72^ program was used to obtain the distribution diagrams. The pH range investigated by using this technique was 2.5–11.0, in accordance with the linear response range of glass electrodes. The solutions were extensively deaerated with an argon flow before the potentiometric runs. A moderate argon flow was maintained throughout the course of the titrations. The different titration curves for each system (at least two) were treated either as a single set or as separate curves without significant variations in the values of the stability constants. Finally, the sets of data were merged and treated simultaneously to give the final stability constants.

### UV–Vis Measurements

The solvents used were of spectroscopic or equivalent grade. Water was twice distilled and passed through a Millipore apparatus. The pH values were measured with a Metrohm 713 pH meter, and adjustments of the hydrogen-ion concentration of the solutions were made with diluted HCl and NaOH solutions. UV–vis absorption spectra were recorded between pH 2 and 11 with an Agilent 8453 spectrometer. The resulting data from the titrations were used to calculate the third protonation constant of ^**COOMe**^ **PyNMe**_**3**_. For this, the HypSpec program was employed, considering all of the absorbance intensities in the range of 250 and 350 nm.^71^

### Electrochemical Measurements

Cyclic (CV) and square wave (SWV) voltammetric experiments were performed with 10^–3^ M aqueous solutions of ^**COOH**^ **PyNH**_**3**_, ^**COOH**^ **PyNMe**_**3**_ at pH = 7.4 in 50 mM TRIS buffer and 0.15 M NaClO4 as the electrolyte. For the study of the electrochemistry of the metal complexes, 0.5 molar amounts of Cu­(ClO_4_)_2_·6H_2_O and/or FeSO_4_·7H_2_O and the ligand were dissolved in 50 mM TRIS. Experiments combined an air-saturated solution with progressively deaerated solutions by bubbling Ar. The experiments were carried out at 298 K using a glassy carbon working electrode (geometrical area 0.071 cm^2^) in a conventional three-electrode cell completed with a Pt wire auxiliary electrode and a Ag/AgCl (3 M NaCl) reference electrode. A CH 660c potentiostatic device was used.

### *In Vitro* McCord–Fridovich SOD Activity Assays

The SOD-like activity was determined by using the nitroblue tetrazolium (NBT) method, which despite its limitations is frequently used to estimate the activity of SOD mimetics.^60–63^ The assays were carried out in a pH = 7.4 50 mM HEPES buffer at 298 K. The xanthine (2.2 × 10^–4^ M) and xanthine oxidase (ca. 3 UN) system was used to generate a reproducible and constant flux of superoxide anions. The rate of reduction of NBT (7.3 × 10^–5^ M) to blue formazan was followed spectrophotometrically at 560 nm. Data in the absence of the complex were used as a reference. The rate of NBT reduction was progressively inhibited after the addition of the complex solutions at increasing concentrations (typically from 0.01 to 5 μM in a cuvette for Cu^2+^ and from 0.1 to 100 μM for Fe^2+^ complexes) prepared in 50 mM Tris-HCl buffer. The percentage of inhibition of the NBT reduction to formazan was used as a measure of the SOD activity of the compounds. The concentration of the complex required to yield 50% inhibition of NBT reduction (IC_50_) was determined from a plot of percentage inhibition versus complex concentration. The IC_50_ data were calculated from the mean values of (at least) three independent measurements. The catalytic constant was calculated from the IC_50_ using the equation *k*_cat_ = *k*_NBT_[NBT]/IC_50_ where *k*_NBT_ = (5.94 ± 0.5) × 10^4^ M^–1^ s^–1^.^73–75^

Caution: Given the prion-like properties of misfolded SOD1 and its potential for self-propagation and transmission between cells, all procedures involving its handling should be conducted under appropriate biosafety precautions to avoid inadvertent seeding or contamination.

### Pressure Measurements

The quantification of the evolved oxygen is frequently used to evaluate the catalase activity.^76–79^ In this work, the amount of gas evolved during the reaction of the Fe^2+^–^**COOH**^ **PyNMe**_**3**_ complex with hydrogen peroxide was monitored by measuring the pressure changes over time using a previously calibrated Man on the Moon X104 gas evolution kit. For that purpose, 2.5 mL of an aqueous solution of the complex at concentrations of (0.5–2.7) × 10^–4^ M buffered at pH 7.5 with TRIS (50 mM) was prepared inside a glovebox, placed in a closed reaction vessel, and purged with nitrogen. Once the pressure readings were stabilized, 0.2–1.2 mL of a 0.12 M aqueous solution of H_2_O_2_ (also prepared in TRIS 50 mM at pH 7.4) was added, and the pressure was then measured as a function of time. A blank experiment was conducted with a complex-free solution of a 50 mM TRIS solution with hydrogen peroxide at pH 7.4, where no O_2_ evolution was detected. The same result was obtained for blank experiments, including also the ligand but not the metal ion or the free metal ion (i.e., Fe^2+^, Fe^3+^, or Cu^2+^) in the absence of the ligand (see Entries 8–12 in Table 5). These results indicate that there is no significant direct oxidation with H_2_O_2_ of the ligand or the buffering agent under the present experimental conditions. Nevertheless, the possible formation of small amounts of CO_2_, which would introduce a small error in the estimation of evolved O_2_, cannot be completely ruled out.

## Supplementary Material



## Notes

Interaction studies with Cu^2+^ and Fe^2+^, complexation constants, distribution diagrams, ^1^H NMR, ^13^C NMR, UV–vis spectra, and fitting of the SOD assays and pressure change representations during the catalase activity determination (PDF)

## ∇

Departament de Química Física, Universitat de València, C/Dr Moliner s/n, 46100 Burjassot, Spain
