# Supplementary material for: Tailoring Antioxidant Activities: Metal-Type Dependent, Highly Active SOD or Catalase Mimetics
Source: Inorg Chem. 2025 Sep 9;64(37):18938–49. doi: 10.1021/acs.inorgchem.5c02973 (PMC12458691; doi:10.1021/acs.inorgchem.5c02973)
Supplement: Supplementary file 1 [file ic5c02973_si_001.pdf]

Supporting Information

Tailoring antioxidant activities: Metal-type dependent, highly active SOD or catalase mimetics

Álvaro Martínez-Camarena,[a,b,c,*] Pablo Navarro-Madramany,[a] Carmen E. Castillo,[d] Antonio Doménech-Carbó,[e] Manuel G. Basallote,[d] Peter Faller,[b,f] Enrique García-España[a,*]

a) ICMol, Departament de Química Inorgànica, Universitat de València. C/ Catedrático José Beltrán 2, 46980, Paterna, Spain.

b) Institut de Chimie, UMR 7177, Université de Strasbourg, CNRS, 4 Rue Blaise Pascal, 67000 Strasbourg, France.

c) MatMoPol Research Group, Department of Inorganic Chemistry, Faculty of Chemical Sciences, Complutense University of Madrid, Avda. Complutense s/n, Madrid, Spain.

d) Departamento de Ciencia de los Materiales e Ingeniería Metalúrgica y Química Inorgánica, Facultad de Ciencias, Instituto de Biomoléculas (INBIO), Universidad de Cádiz, Puerto Real, 11510 Cádiz, Spain.

e) Departament de Química Analítica, Universitat de València. C/ Dr Moliner s/n, 46100 Burjassot, Spain.

f) Institut Universitaire de France (IUF), 1 rue Descartes, 75231 Paris, France.

§ Current affiliation: Departament de Química Física, Universitat de València. C/ Dr Moliner s/n, 46100 Burjassot, Spain.

* To whom correspondence should be addressed. E-mail: alvaro.martinez@uv.es (Á. Martínez-Camarena) and enrique.garcia-es@uv.es (E. García-España).

Table of contents

I. Interaction with Cu^{2+} and Fe^{2+} ions........3

II. Other figures........5

III. References........20

I. Interaction with Cu^{2+} and Fe^{2+} ions

The distribution diagrams have enabled the calculation of pCu and pFe values at physiological pH 7.4 in the presence of the different ligands for $[L]=[Cu^{2+}]=[Fe^{2+}]=1$ μM and 10 μM solutions. The results of the determinations can be found in Table S1. The pCu values were found to be between 8.68 to 9.80 both for concentrations of all the species of 1 and 10 μM, indicating that Cu^{2+} is completely coordinated at pH 7.4 in the presence of any of the studied ligands. However, for Fe^{2+}, the logarithmic values of the concentration of free ions significantly diminish to values ranging *ca.* 6.2 for 1 μM concentration of the studied species, and *ca.* 5.6 for 10 μM concentrations. This suggests that part of the Fe^{2+} will be free in solution at pH 7.4 for highly diluted solutions, a significant portion will still be coordinated to the ligands, allowing their performance as ROS scavengers.

Table S1. pCu and pFe values of the ligands calculated at pH 7.4 for theoretical solutions containing 10^{-5} or 10^{-6} M of L and Cu^{2+} or Fe^{2+}.

[L]=[M]=1 μM	^{COOH}**PyNH$_3$**	^{COOH}**PyNMe$_3$**	^{COOMe}**PyNMe$_3$**
pCu	9.80	9.18	9.79
pFe	-	6.22	6.27
[L]=[M]=10 μM	^{COOH}**PyNH$_3$**	^{COOH}**PyNMe$_3$**	^{COOMe}**PyNMe$_3$**
pCu	9.30	8.68	9.29
pFe	-	5.58	5.66

Regarding the comparison between the Cu^{2+} coordination determined for ^{COOH}**PyNH$_3$**, ^{COOH}**PyNMe$_3$** and ^{COOMe}**PyNMe$_3$** and those reported previously for the pyridine azamacrocyclic analogues shown in Figure S1, there is not a significant difference in their stability constants. Indeed, while the formation constant for the mononuclear $[CuL]^{(2+x)+}$ complex is 17.62 logarithmic units for ^{COOH}**PyNH$_3$** and 16.35 for ^{COOH}**PyNMe$_3$**, those reported for the equivalent complexes of ^{H}**PyNH$_3$** and ^{H}**PyNMe$_3$** present a value of 17.78 and 16.44 logarithmic units, respectively (see Tables S2 and S3).[1] This implies a difference of just *ca.* 0.1 logarithmic units in both cases.

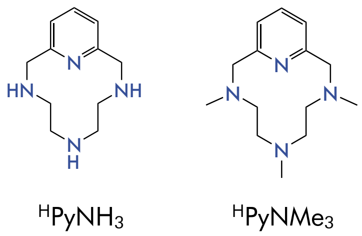

Figure S1. Azamacrocyclic analogues reported in bibliography.[1]

Table S2. Logarithms of the stepwise protonation constants for **^{H}PyH$_3$** and **^{H}PyMe$_3$** obtained by potentiometry.[1] The constants were determined in 0.15 M $NaClO_4$ at 298.1 ± 0.1 K

Reaction	**^{H}PyNH$_3$**	**^{H}PyNMe$_3$**
$H + L \rightleftarrows HL$	10.54(1) [a]	10.88(1)
$H + HL \rightleftarrows H_2L$	7.96(1)	7.37(1)
$H + H_2L \rightleftarrows H_3L$	1.90(1)	-
**log β**[b]	20.40	18.25

a) Values in parentheses are standard deviations in the last significant figure. b) $\text{Log}\,\beta = \sum \log K$

Table S3. Logarithms of the stepwise stability constants for the Cu^{2+} and Fe^{2+} complexes of **^{H}PyNH$_3$** and **^{H}PyNMe$_3$** obtained by potentiometric measurements.[1] The constants were determined in 0.15 M $NaClO_4$ at 298.1 ± 0.1 K.

Reaction	**^{H}PyNH$_3$**	**^{H}PyNMe$_3$**
$Cu^{2+} + L \rightleftarrows [CuL]^{2+}$	17.78(2) [a]	16.44(3)
$[CuL]^{2+} + H_2O \rightleftarrows [CuL(OH)]^{+} + H^{+}$	-8.68(8)	-8.53(4)
$[CuL(OH)]^{+} + H_2O \rightleftarrows [CuL(OH)_2]^{+} + H^{+}$	-10.7(1)	-11.31(8)
$Fe^{2+} + L \rightleftarrows [FeL]^{2+}$	12.68(2)	9.72(2)
$[FeL]^{2+} + H_2O \rightleftarrows [FeL(OH)] + H^{+}$	-6.94(5)	-9.08(4)
$[FeL(OH)]^{+} + H_2O \rightleftarrows [FeL(OH)_2]^{+} + H^{+}$	-	-10.58(5)

a) Values in parentheses are standard deviations in the last significant figure.

II. Other figures

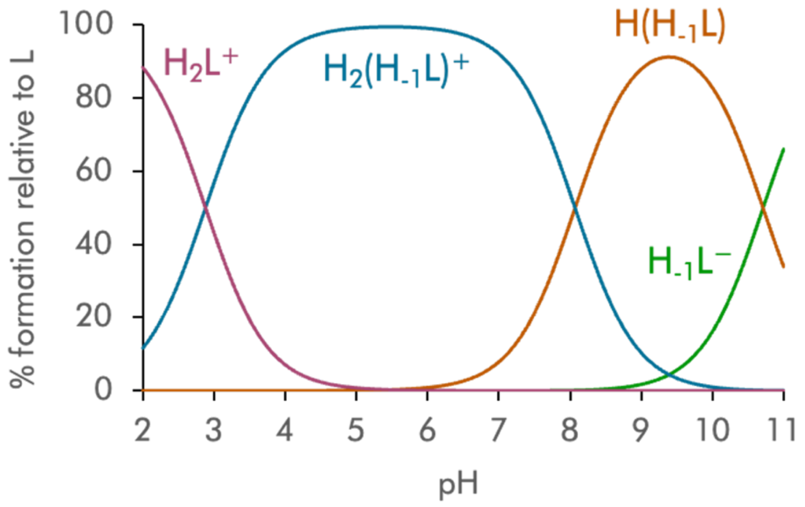

Figure S2. Distribution diagram of **^{COOH}PyNH$_3$** as a function of the pH in aqueous solution (0.15 M NaClO$_4$, 298.1 ± 0.1 K). Taken from reference 2.

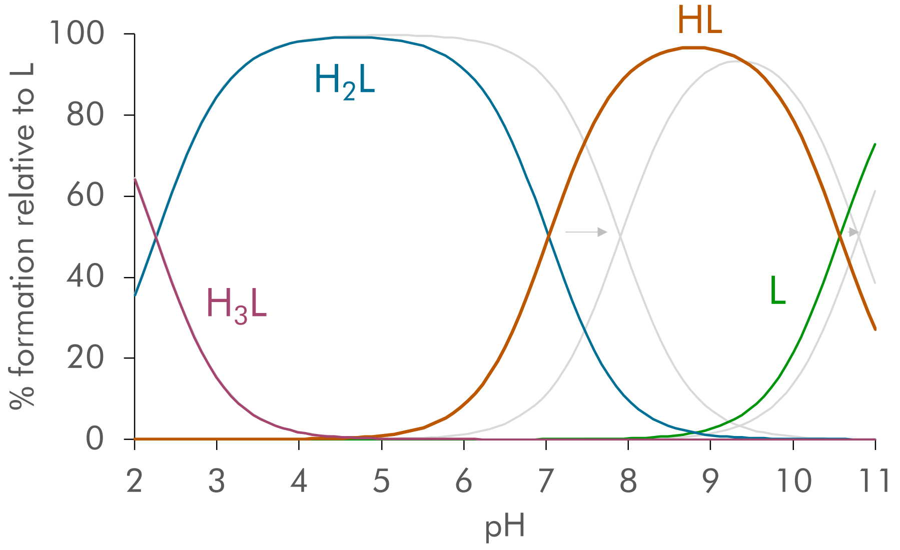

Figure S3. Comparison between the distribution diagrams of **^{COOMe}PyNMe$_3$** (represented as L) and **^{COOH}PyNH$_3$** as a function of the pH in aqueous solution. The coloured lines correspond to **^{COOMe}PyNMe$_3$** while the grey ones stand for the equivalent species for **^{COOH}PyNH$_3$**. [**^{COOMe}PyNMe$_3$**]=[**^{COOH}PyNH$_3$**]=10^{-3} M. The charges have been omitted for clarity.

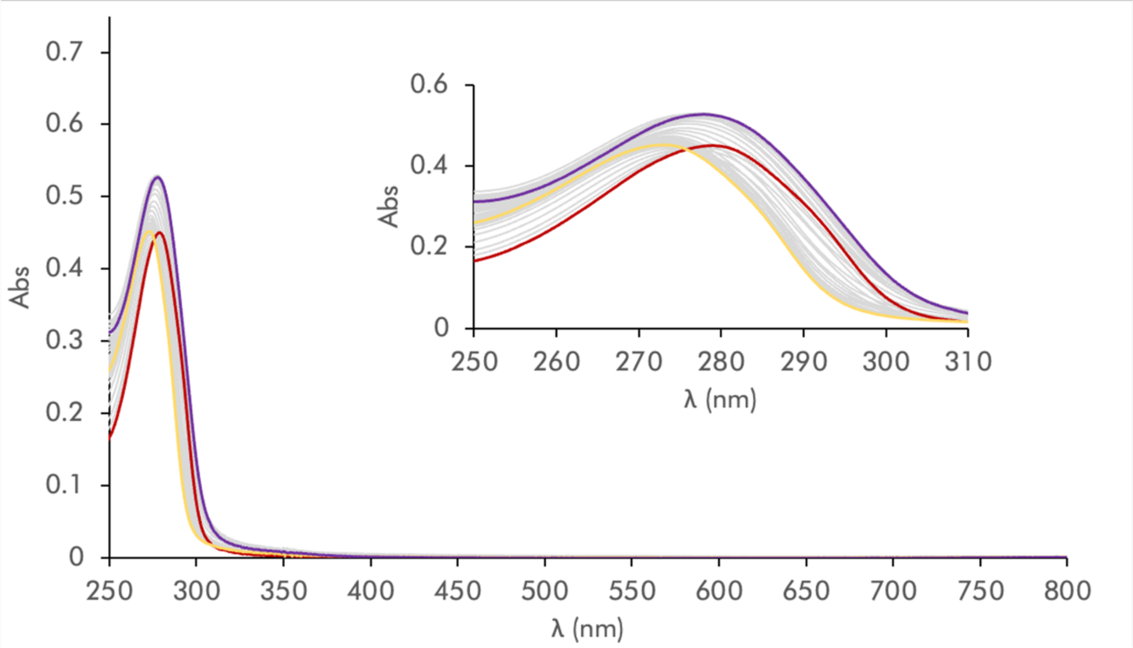

Figure S4. Spectra of a 10^{-4} M solution of ^{COOH}**PyNMe**$_3$ at different pH values (from 1.98 11.24). The spectra corresponding to the inflexion points have been represented in a different colour: red for the spectrum at pH 1.98, yellow for the one at pH 4.04 and purple for the one at pH 9.09.

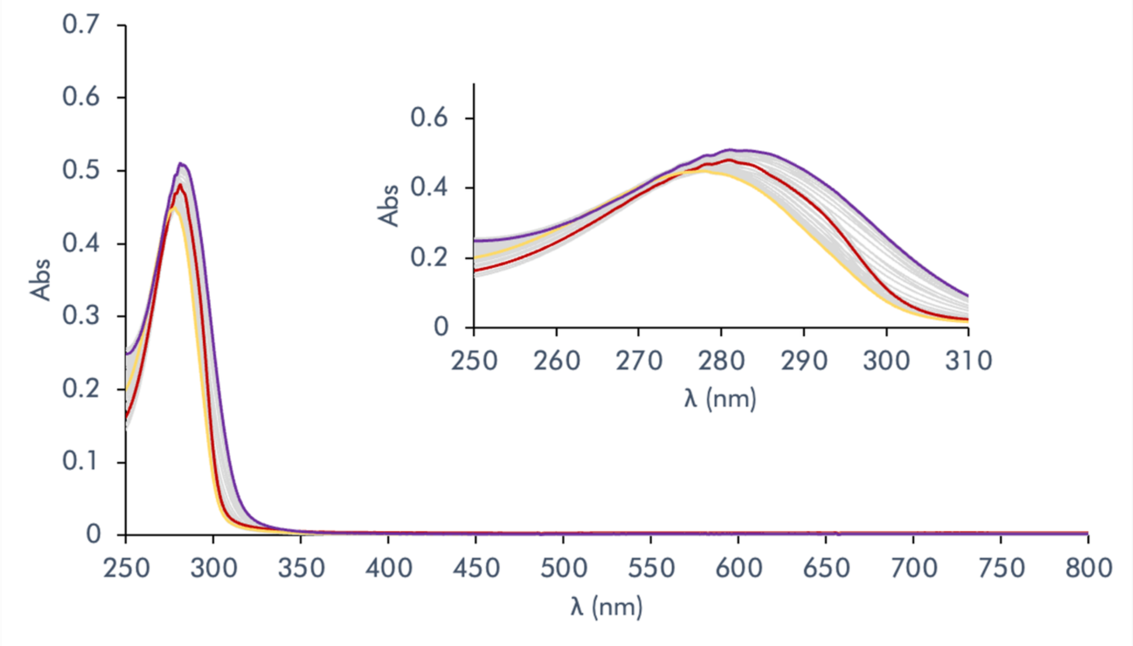

Figure S5. Spectra of a 10^{-4} M solution of ^{COOMe}**PyNMe**$_3$ at different pH values (from 1.98 11.24). The spectra corresponding to the inflexion points have been represented in a different colour: red for the spectrum at pH 1.98, yellow for the one at pH 4.04 and purple for the one at pH 9.09.

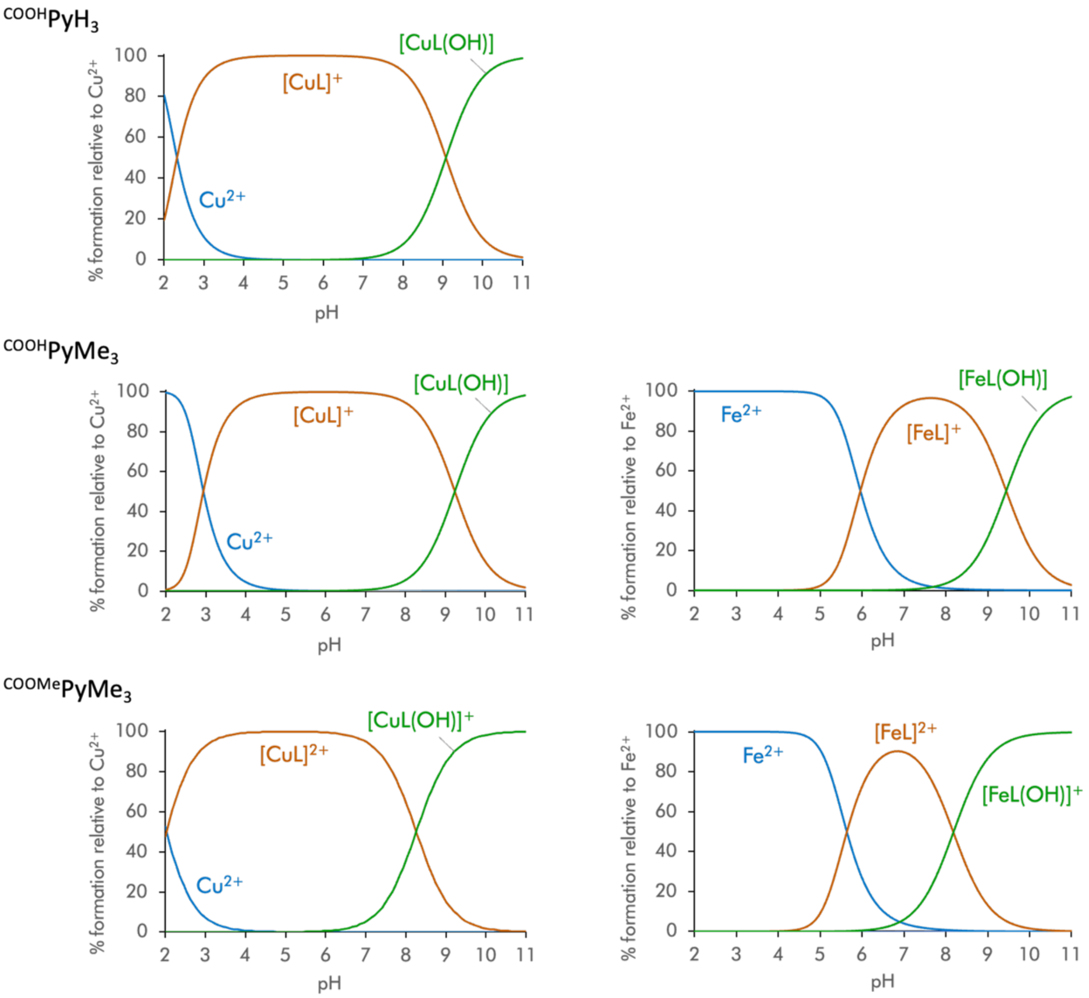

Figure S6. Species distribution curves for the Cu^{2+} (right) and Fe^{2+} (left) complexes in aqueous solution (0.15 M $NaClO_4$, 298.1 ± 0.1 K) of ^{COOH}**PyNH$_3$**, ^{COOH}**PyNMe$_3$** and ^{COOMe}**PyNMe$_3$**. For ^{COOH}**PyNH$_3$** and ^{COOH}**PyNMe$_3$**, L corresponds to the form of the ligand in which the carboxylate is deprotonated. $[Cu^{2+}] = [Fe^{2+}] = [L] = 10^{-3}$ M.

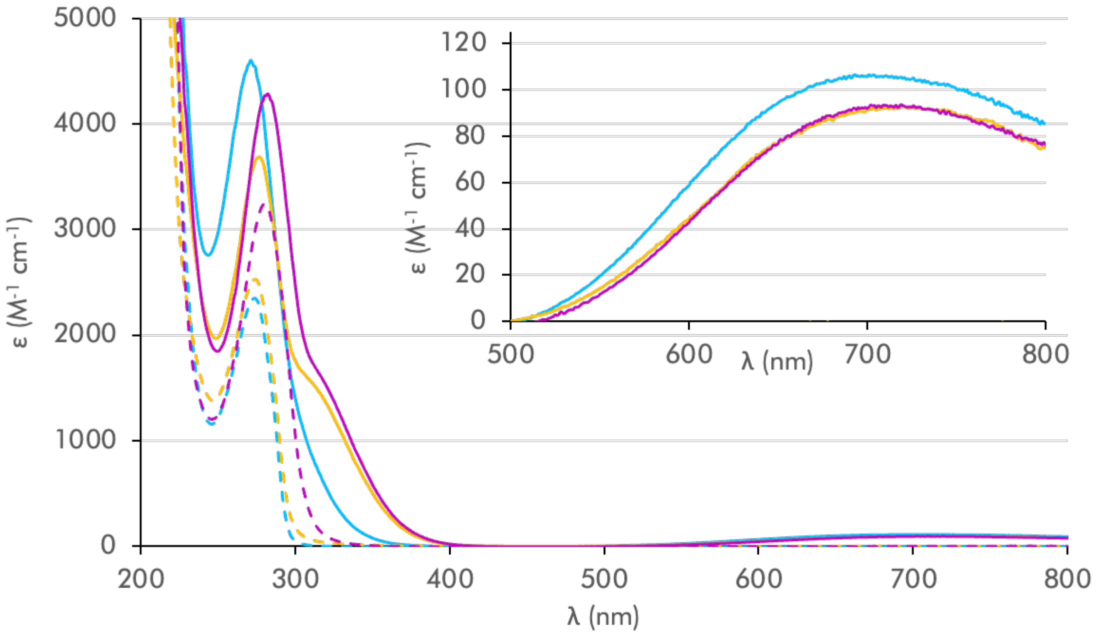

Figure S7. Absorbance spectra of the Cu^{2+} complexes of ^{COOH}**Py22NH3** (blue), ^{COOH}**Py22NMe3** (yellow) and ^{COOMe}**Py22NMe3** (purple). Dashed lines correspond to solutions that do not contain Cu^{2+}. $[L] = [Cu^{2+}] = 5 \times 10^{-4}$ M.

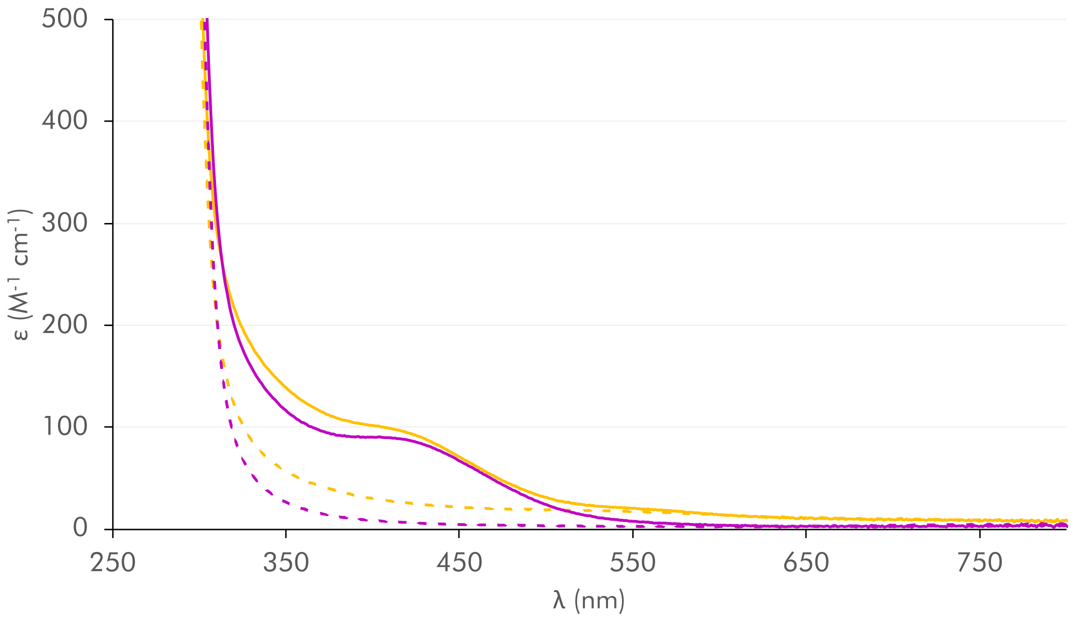

Figure S8. Absorbance spectra of the Fe^{2+} complexes of ^{COOH}**Py22NMe3** (yellow) and ^{COOMe}**Py22NMe3** (purple). Dashed lines correspond to solutions that do not contain Fe^{2+}. $[L] = [Fe^{2+}] = 10^{-3}$ M.

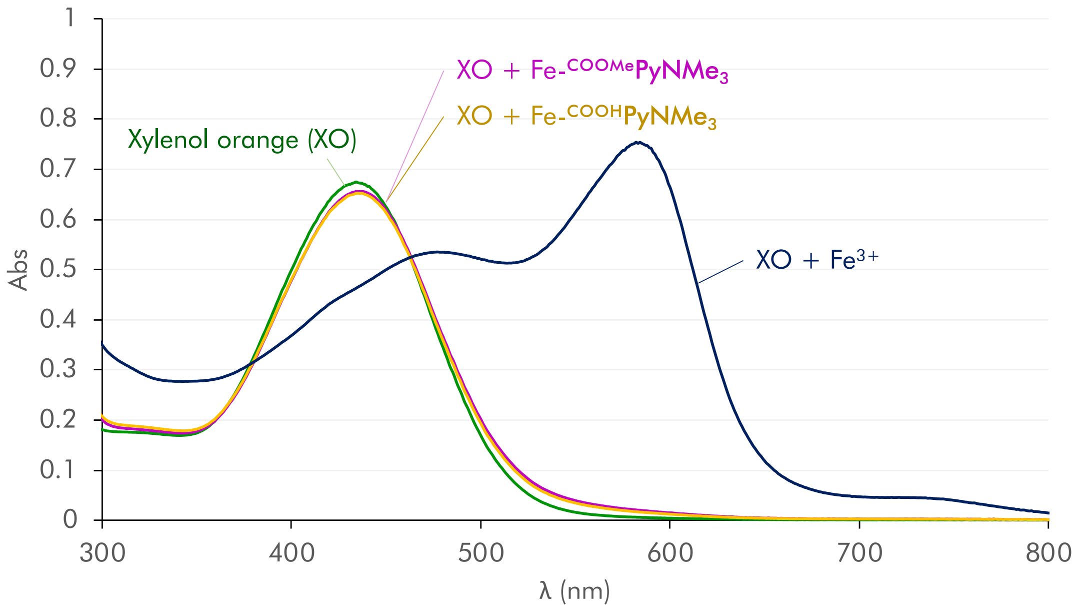

Figure S9. Absorbance spectra of the Fe^{2+} complexes of **^{COOH}Py22NMe$_3$** (yellow) and **^{COOMe}Py22NMe$_3$** (purple) in presence of xylenol orange. For comparison, the spectra of xylenol orange (green) and xylenol orange in presence of Fe^{3+} (dark blue) have been included. [L] = [Fe^{2+}] = 5 ×10^{-5} M; [Fe^{3+}] = 5 ×10^{-5} M; [xylenol orange] = 6 ×10^{-5} M.

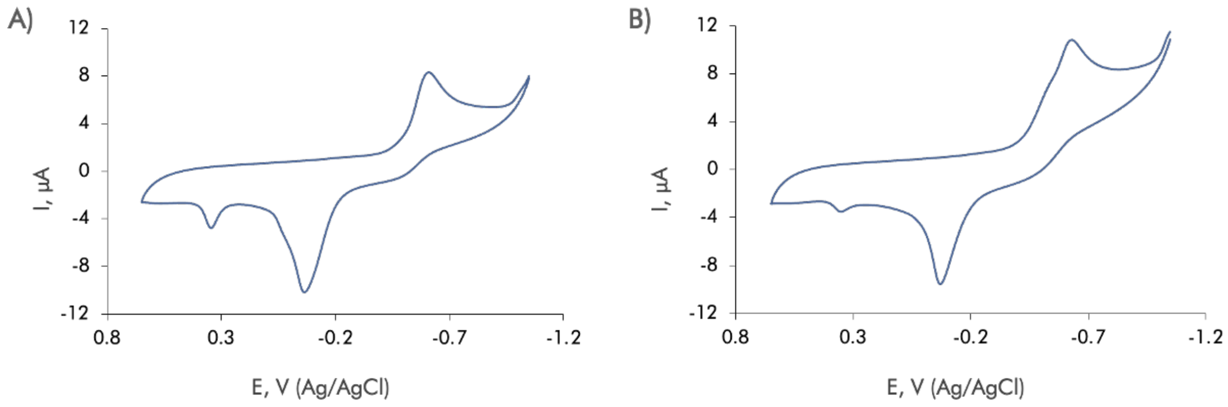

Figure S10. CVs at GCE of a 5 × 10^{-4} M **^{COOH}PyNMe$_3$** plus 2.5 × 10^{-4} M Cu^{2+} solution in TRIS buffer 50 mM pH 7.4. a) deaerated; b) air-saturated. Potential scan initiated at 0.0 V in the negative direction; potential scan rate 50 mV s^{-1}.

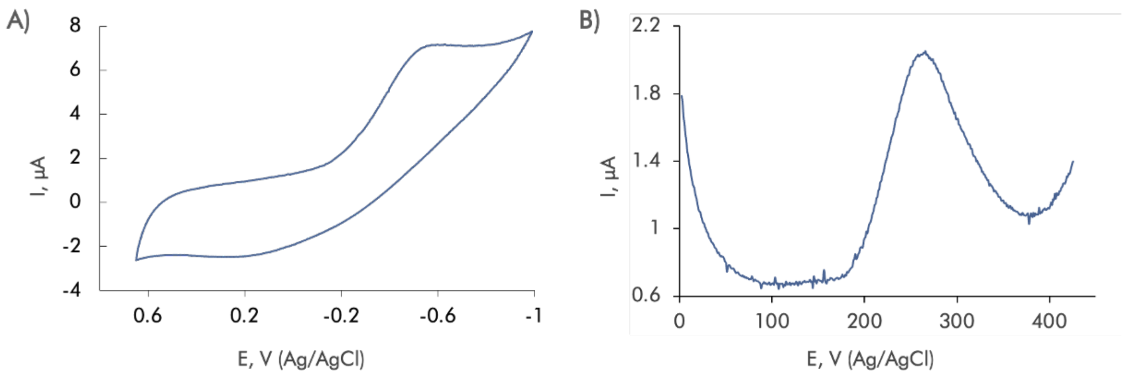

Figure S11. a) CV and b) SWV at GCE of an air-saturated solution of ^{COOH}**PyNMe**$_3$ (10^{-4} M) in TRIS buffer 50 mM pH 7.4. A) Potential scan initiated at 0.0 V in the negative direction; potential scan rate 50 mV s^{-1}. B) Potential scan initiated at 0.65 V in the negative direction; potential step increment 4 mV; square wave amplitude 25 mV; frequency 10 Hz.

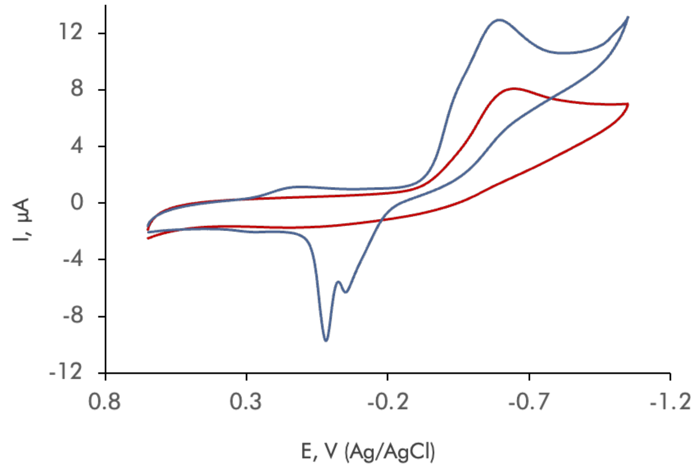

Figure S12. CVs at GCE of an air-saturated 5×10^{-4} M ^{COOH}**PyNMe**$_3$ plus 2.5×10^{-4} M Cu^{2+} plus 2.5×10^{-4} M Fe^{2+} solution in TRIS buffer (blue lines) superimposed to the CV for an air-saturated 10^{-3} M ^{COOH}**PyNMe**$_3$ solution in TRIS buffer (red lines). Potential scan initiated at 0.1 V in the positive direction; potential scan rate 50 mV s^{-1}.

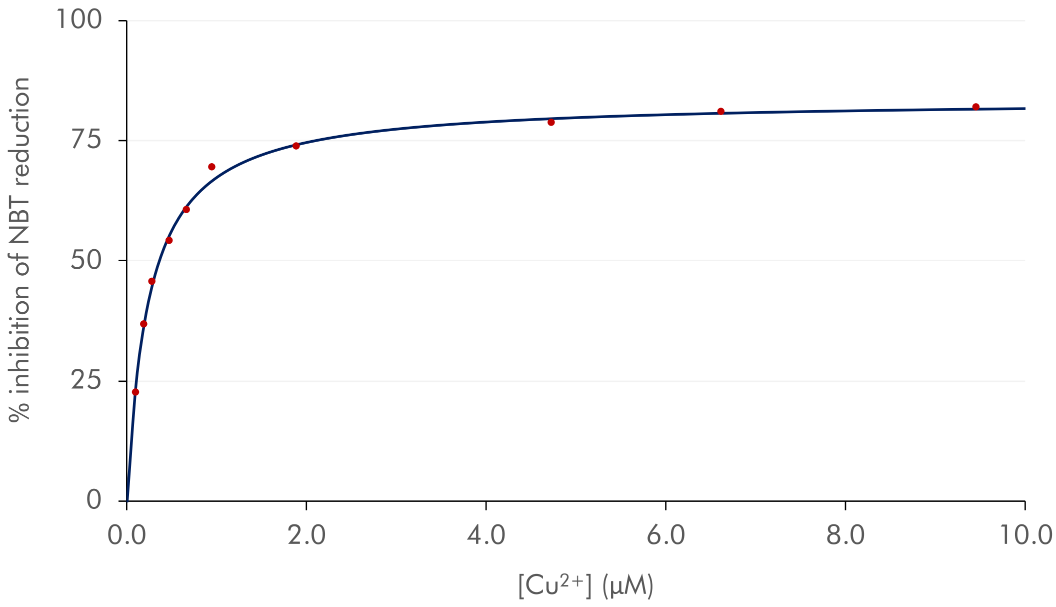

Figure S13. Fitting of the SOD activity data obtained by the McCord-Fridovich method for the system Cu^{2+}-^{COOH}**PyNH**$_3$.

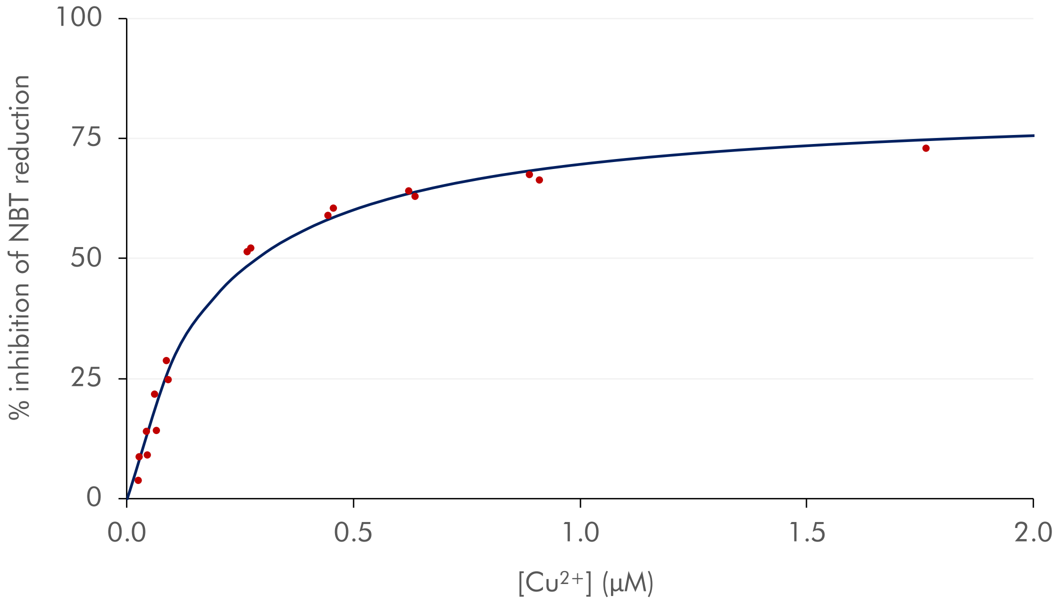

Figure S14. Fitting of the SOD activity data obtained by the McCord-Fridovich method for the system Cu^{2+}-^{COOH}**PyNMe**$_3$.

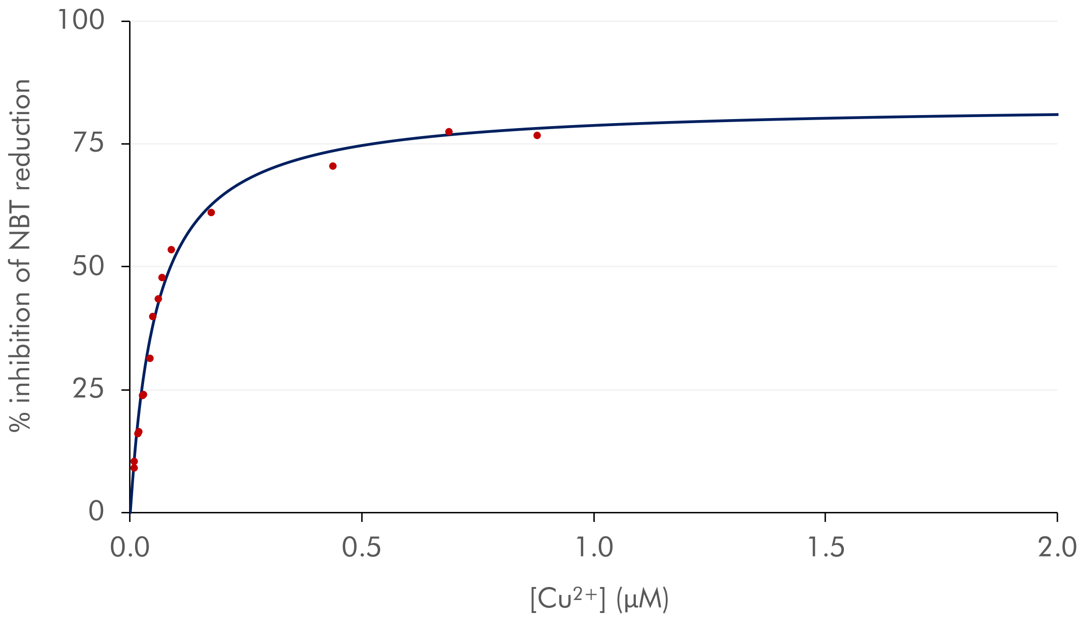

Figure S15. Fitting of the SOD activity data obtained by the McCord-Fridovich method for the system Cu^{2+}-^{COOMe}**PyNMe$_3$**.

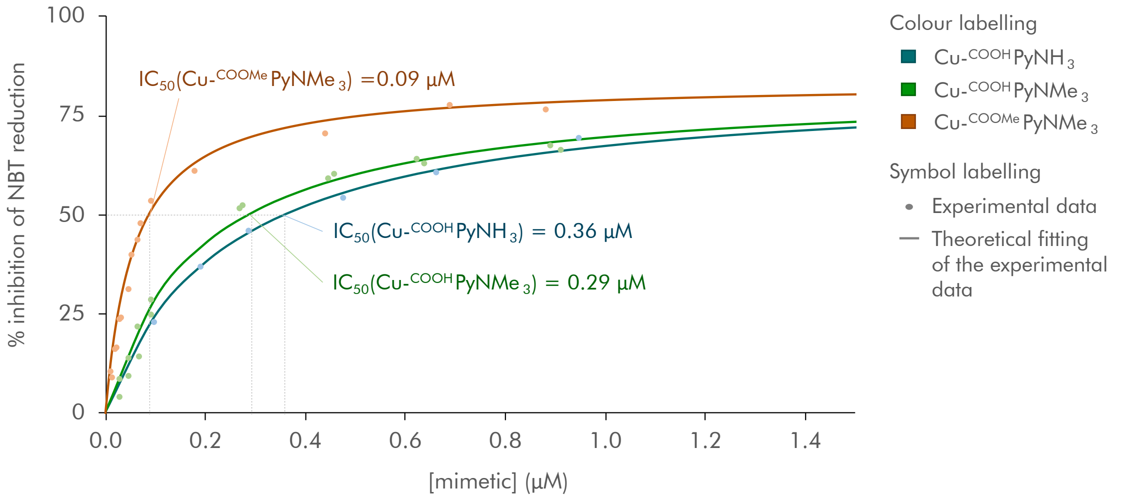

Figure S16. Fitting of the McCord-Fridovich assays as a measurement of the SOD activity. As an indication, the higher the inhibition of NBT reduction for one given concentration of complex, the higher the SOD activity.

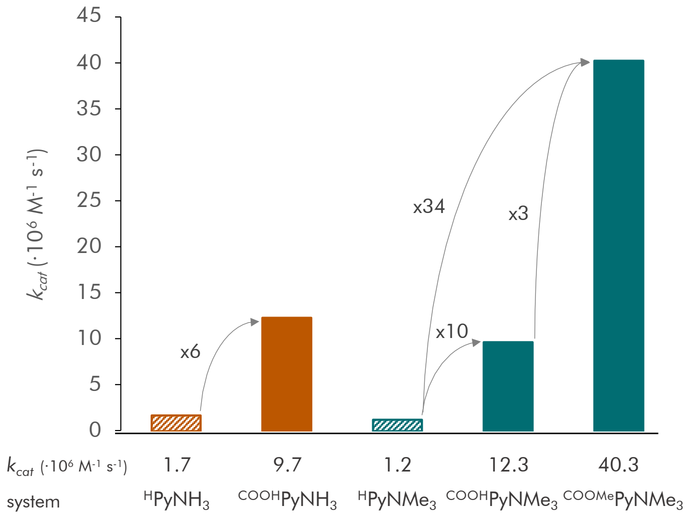

Figure S17. Representation of the catalytic constant values of the systems Cu^{2+} complexes of **^{COOH}PyNH$_3$**, **^{COOH}PyNMe$_3$**, **^{COOHMe}PyNMe$_3$**, and the reference ligands **^{H}PyNH$_3$** and **^{H}PyNMe$_3$**.[1] Introduction of a carboxylic group un *para* to the N in the pyridine in **^{H}PyNH$_3$** and **^{H}PyNMe$_3$** leads to an 6-10 fold increase of the SOD activity of its Cu^{2+} complexes, while introduction of a methyl ester group leads to a 34-fold enhancement.

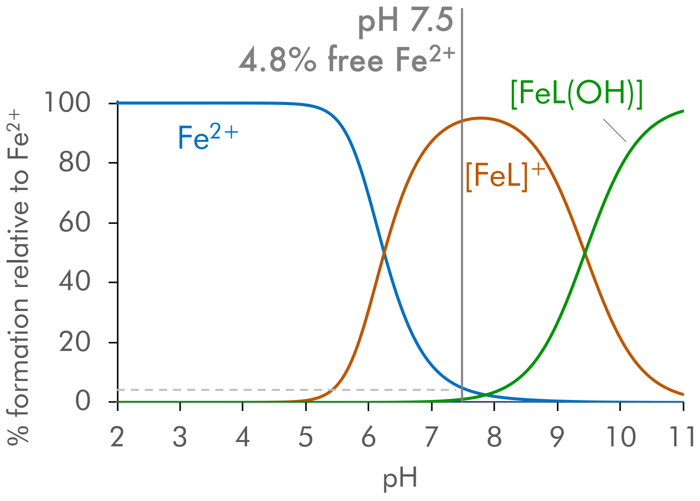

Figure S18. Species distribution curves for the Fe^{2+} complexes in aqueous solution (0.15 M $NaClO_4$, 298.1 ± 0.1 K) of **^{COOH}PyNMe$_3$** at [**^{COOH}PyNMe$_3$**]=[Fe^{2+}]=270 μM.

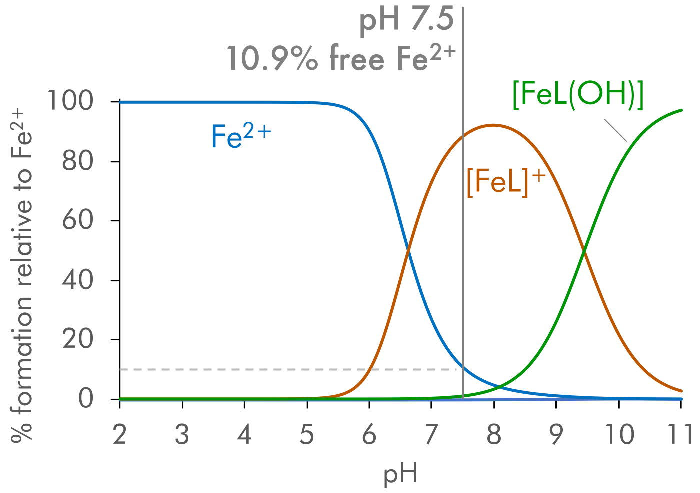

Figure S19. Species distribution curves for the Fe^{2+} complexes in aqueous solution (0.15 M $NaClO_4$, 298.1 ± 0.1 K) of ^{COOH}**PyNMe$_3$** at [^{COOH}**PyNMe$_3$**]=[Fe^{2+}]=50 μM.

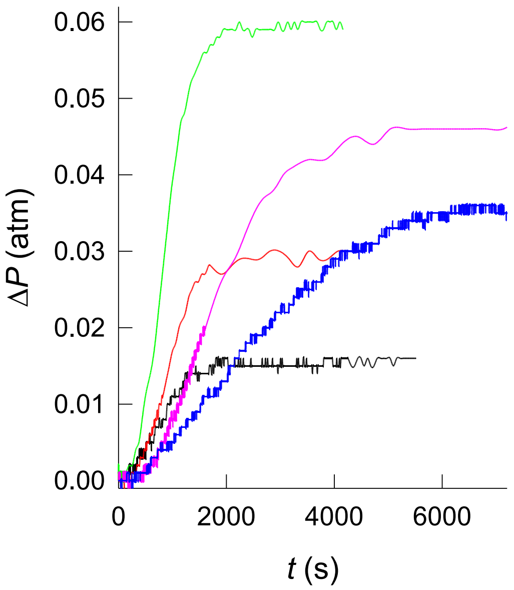

Figure S20. Pressure changes during the reaction of the Fe^{2+}-^{COOH}**PyNMe$_3$** complex with H_2O_2 at pH 7.5 and 25ºC. The concentrations of complex and H_2O_2 are those indicated in Table 5 for entries 1 (black), 2 (red), 3 (green), 4 (pink) and 6 (blue).

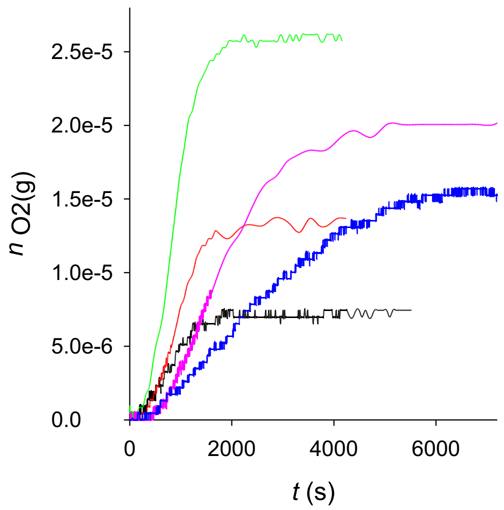

Figure S21. Plot showing the moles of O_2 in the gas phase calculated from the corresponding curves in Figure S20. The concentrations of Fe^{2+}-^{COOH}**PyNMe**$_3$ and H_2O_2 are those indicated in Table 5 for entries 1 (black), 2 (red), 3 (green), 4 (pink) and 5 (blue).

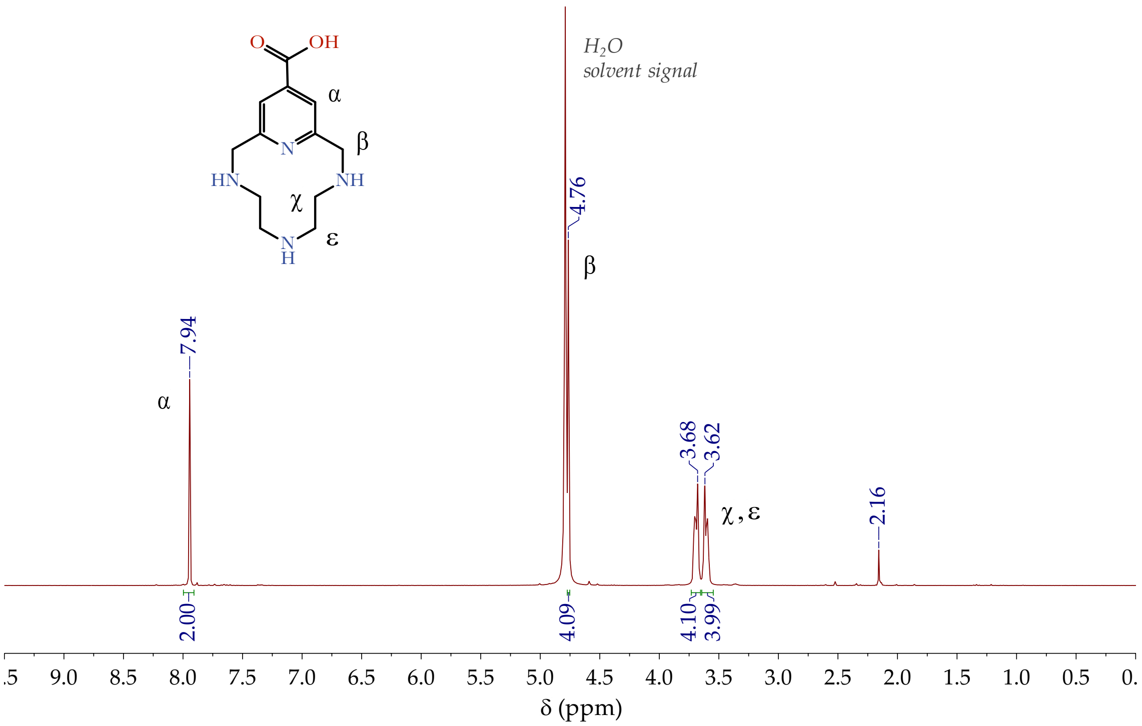

Figure S22. ^{1}H-NMR spectrum of ^{COOH}**PyNH**$_3$ in D_2O at 298 K.

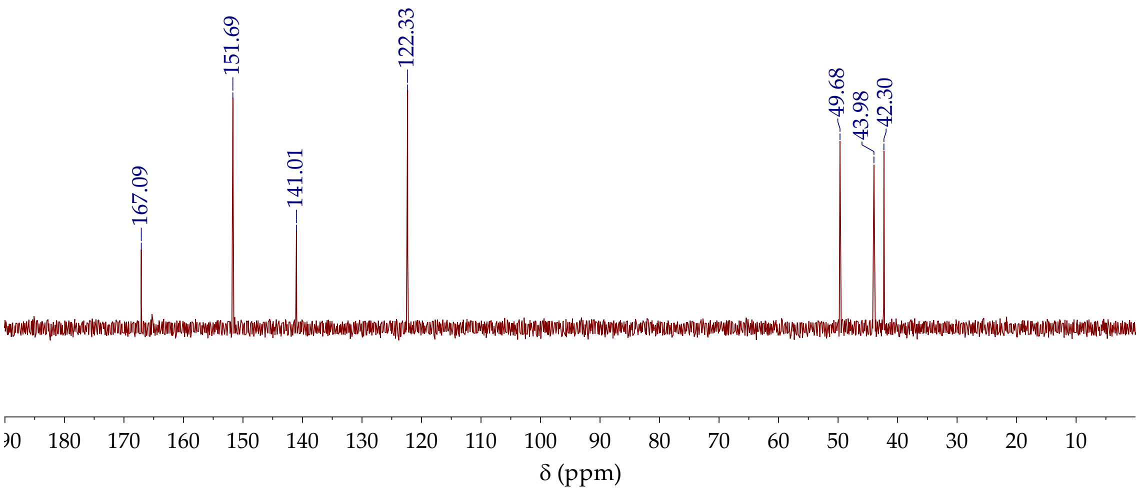

Figure S23. ^{13}C-NMR spectrum of ^{COOH}**PyNH$_3$** in D_2O at 298 K.

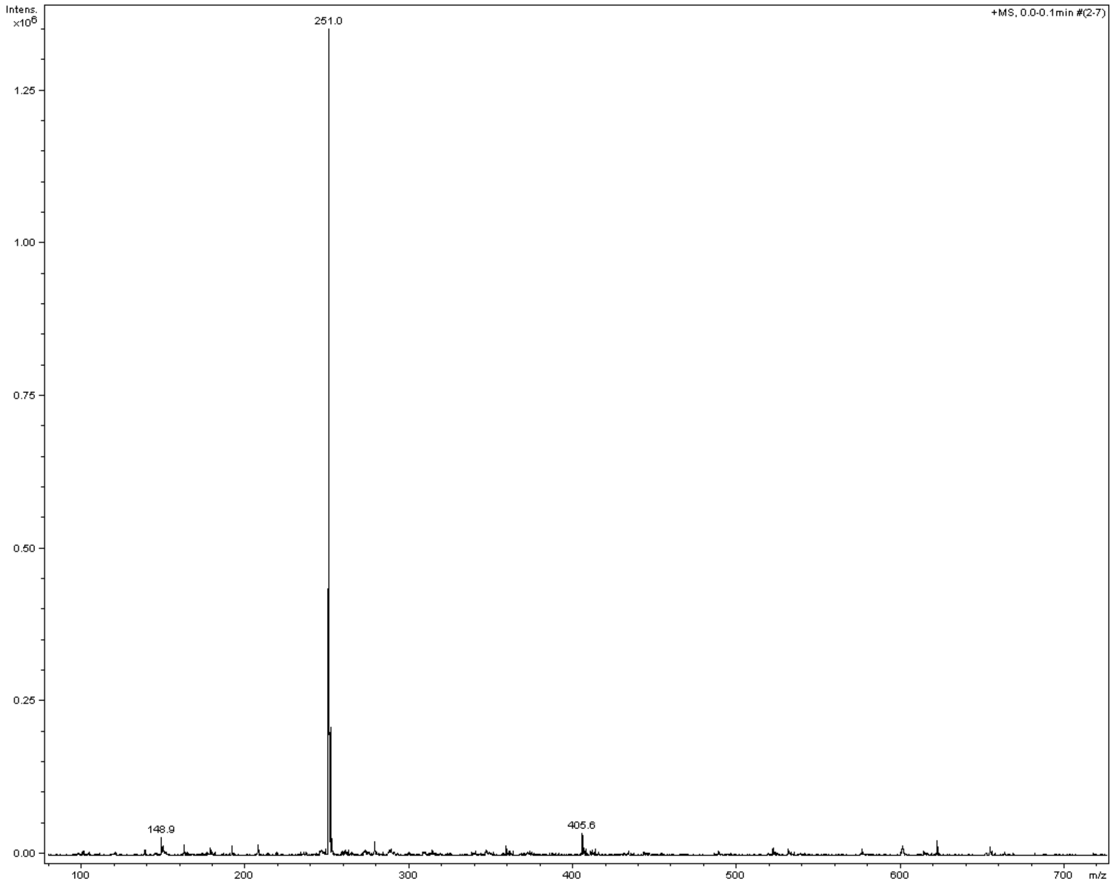

Figure S24. LC-MS (ESI/APCI-TOF) of ^{COOH}**PyNH$_3$** in H_2O.

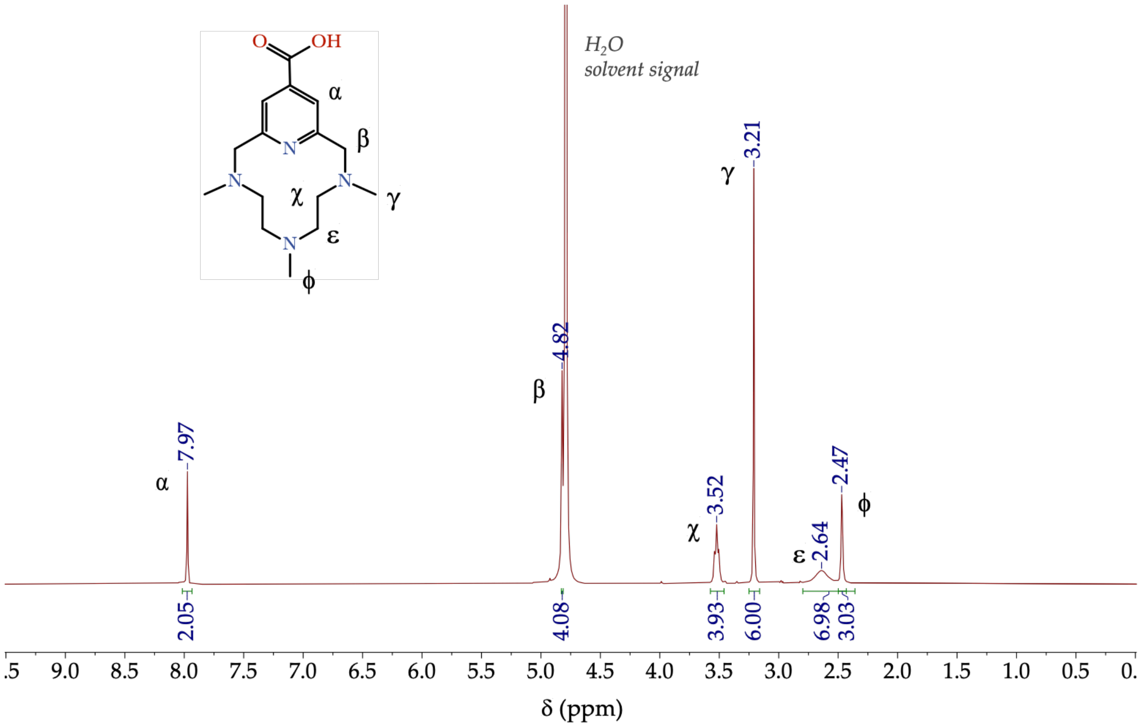

Figure S25. ^{1}H-NMR spectrum of ^{COOH}**PyNMe**$_3$ in D_2O at 298 K.

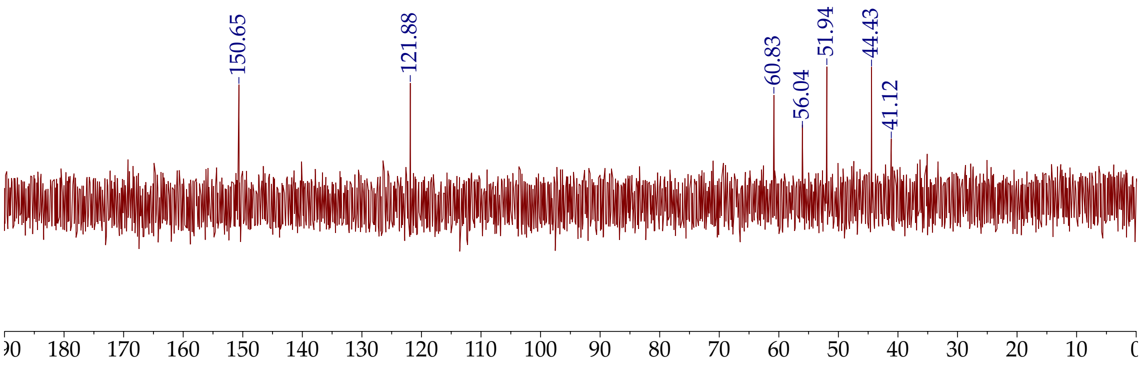

Figure S26. ^{13}C-NMR spectrum of ^{COOH}**PyNMe**$_3$ in D_2O at 298 K.

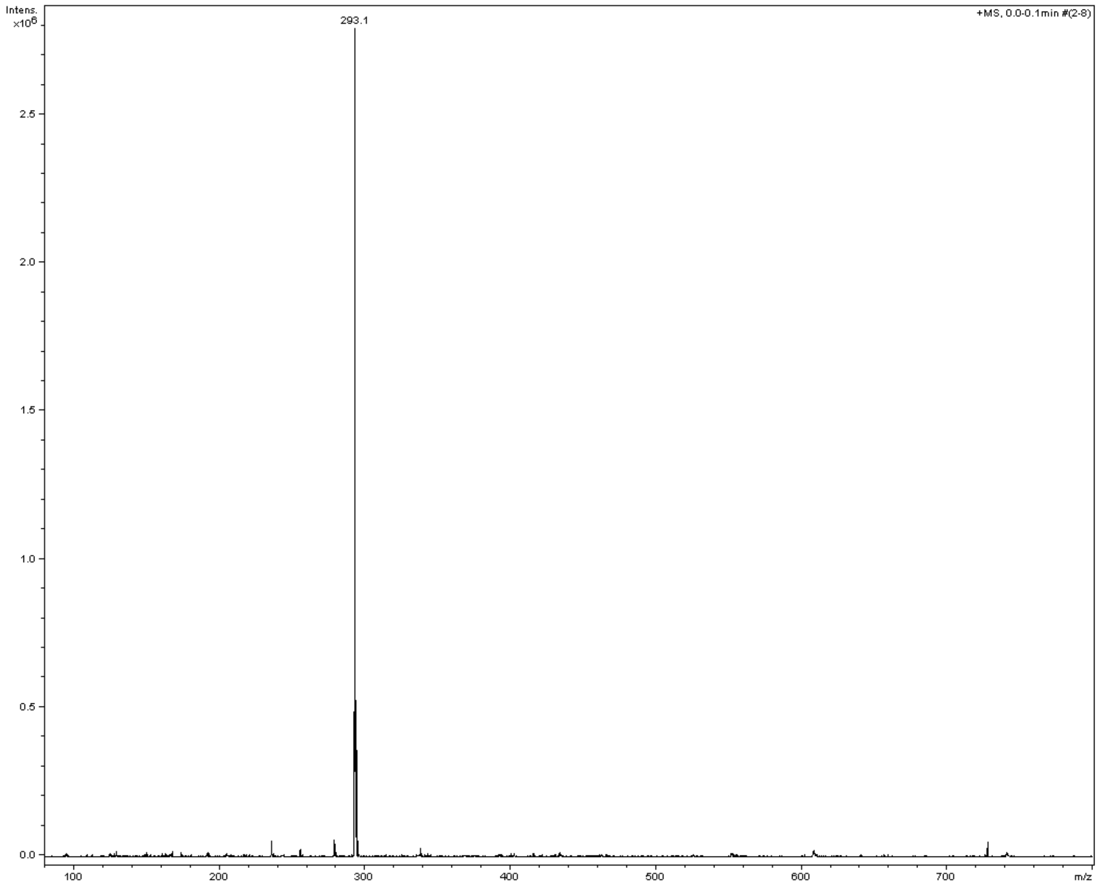

Figure S27. LC-MS (ESI/APCI-TOF) of ^{COOH}**PyNMe**$_3$ in H_2O.

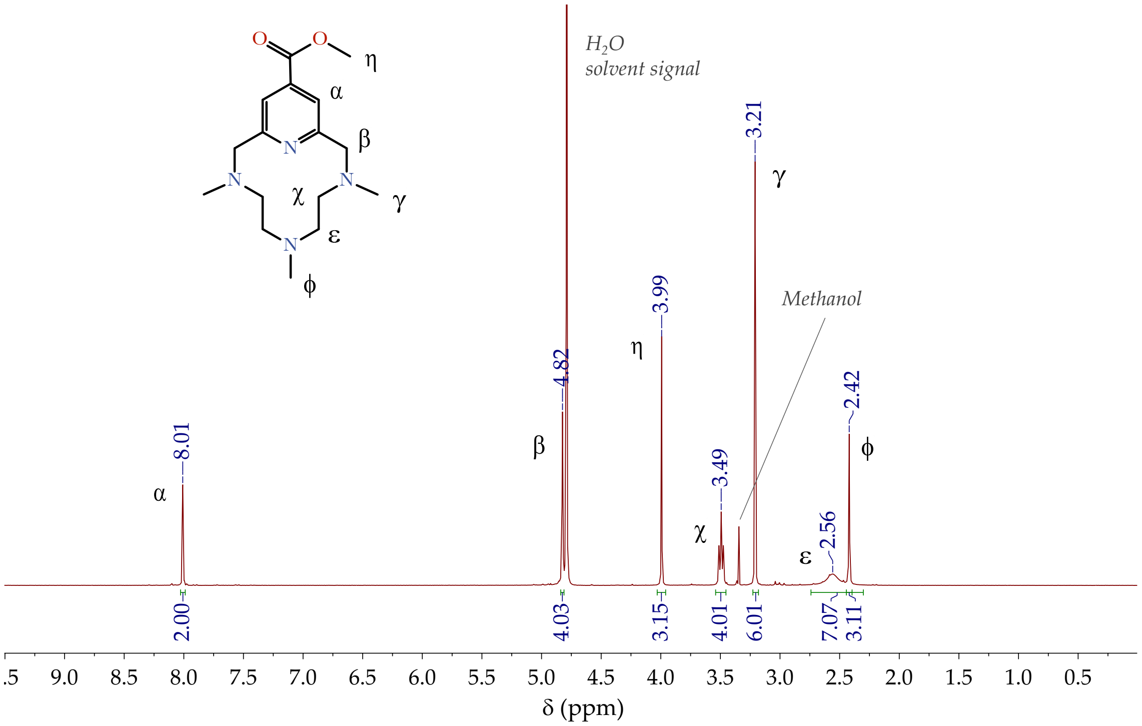

Figure S28. ^{1}H-NMR spectrum of ^{COOMe}**PyNMe**$_3$ in D_2O at 298 K.

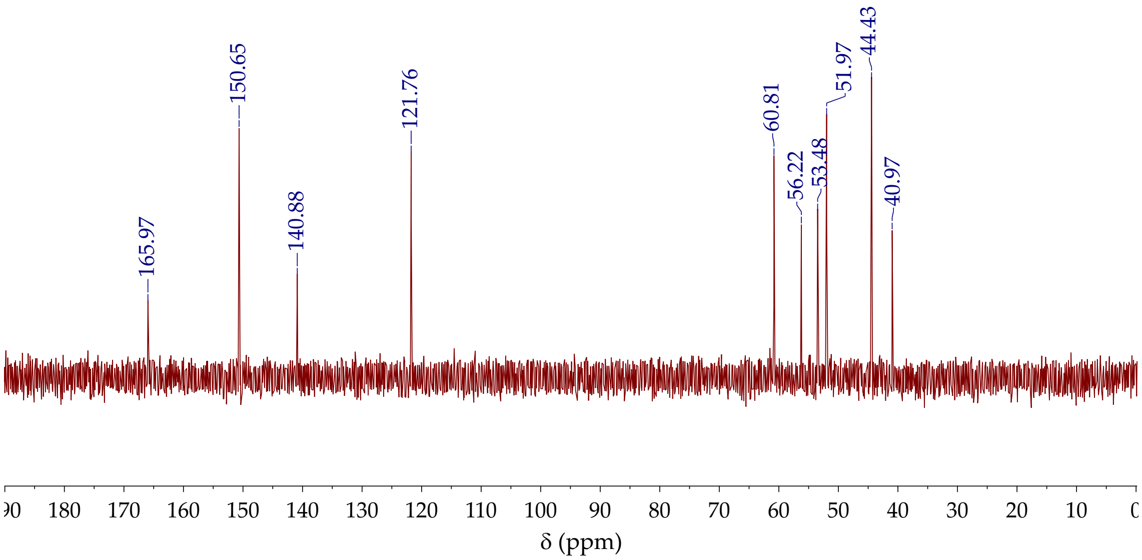

Figure S29. ^{13}C-NMR spectrum of ^{COOMe}**PyNMe**$_3$ in D_2O at 298 K.

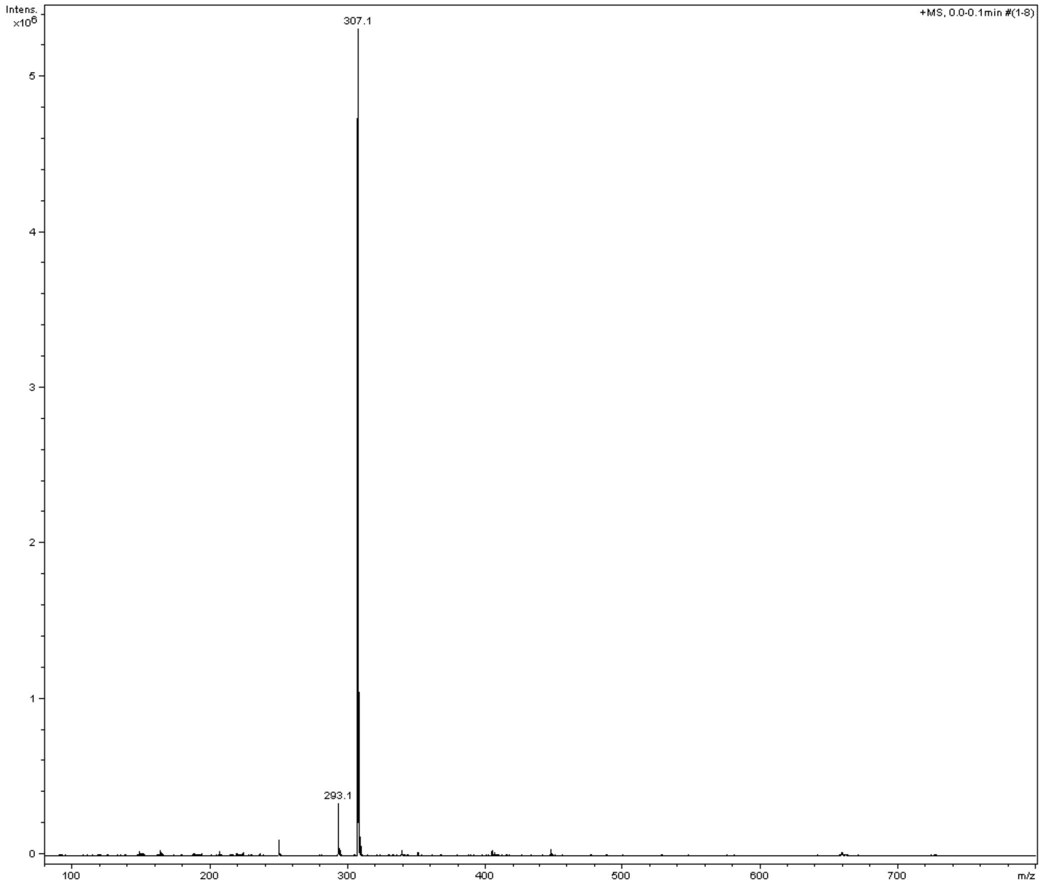

Figure S30. LC-MS (ESI/APCI-TOF) of ^{COOMe}**PyNMe**$_3$ in H_2O.

III. References

1 Martínez-Camarena, Á.; Liberato, A.; Delgado-Pinar, E.; Algarra, A. G.; Pitarch-Jarque, J.; Llinares, J. M.; Mañez, M. Á.; Domenech-Carbó, A.; Basallote, M. G.; García-España, E. Coordination Chemistry of Cu^{2+} Complexes of Small N-Alkylated Tetra-Azacyclophanes with SOD Activity. *Inorg. Chem.*, **2018**, *57*, 10961–10973. DOI: 10.1021/acs.inorgchem.8b01492.

2 Martínez-Camarena, Á.; Merino, M.; Sánchez-Sánchez, A. V.; Blasco, S.; Llinares, J. M.; Mullor, J. L.; García-España, E. An Antioxidant Boehmite Amino-Nanozyme Able to Disaggregate Huntington's Inclusion Bodies. *Chem. Commun.* **2022**, *58* (32), 5021–5024. DOI: 10.1039/D2CC01257J.

3 Mekhail, M. A.; Smith, K. J.; Freire, D. M.; Pota, K.; Nguyen, N.; Burnett, M. E.; Green, K. N. Increased Efficiency of a Functional SOD Mimic Achieved with Pyridine Modification on a Pyclen-Based Copper(II) Complex. *Inorg. Chem.*, **2023**, *62*, 5415-5425. DOI: 10.1021/acs.inorgchem.2c04327.
